# Fractalkine (CX_3_CL1) enhances hippocampal *N*-methyl-d-aspartate receptor (NMDAR) function via d-serine and adenosine receptor type A2 (A_2A_R) activity

**DOI:** 10.1186/1742-2094-10-108

**Published:** 2013-08-27

**Authors:** Maria Scianni, Letizia Antonilli, Giuseppina Chece, Gloria Cristalli, Maria Amalia Di Castro, Cristina Limatola, Laura Maggi

**Affiliations:** 1 Institute Pasteur-Cenci Bolognetti Foundation, Department of Physiology and Pharmacology, University Sapienza, Rome, Italy; 2 School of Pharmacy, Medicinal Chemistry Unit, University of Camerino, Camerino, Italy; 3 IRCCS Neuromed, Pozzilli, Italy

**Keywords:** CX_3_CL1, NMDAR, Microglia, Hippocampus, d-serine, Adenosine receptors

## Abstract

**Background:**

*N*-Methyl-d-aspartate receptors (NMDARs) play fundamental roles in basic brain functions such as excitatory neurotransmission and learning and memory processes. Their function is largely regulated by factors released by glial cells, including the coagonist d-serine. We investigated whether the activation of microglial CX_3_CR1 induces the release of factors that modulate NMDAR functions.

**Methods:**

We recorded the NMDAR component of the field excitatory postsynaptic potentials (NMDA-fEPSPs) elicited in the CA1 stratum radiatum of mouse hippocampal slices by Shaffer collateral stimulation and evaluated d-serine content in the extracellular medium of glial primary cultures by mass spectrometry analysis.

**Results:**

We demonstrated that CX_3_CL1 increases NMDA-fEPSPs by a mechanism involving the activity of the adenosine receptor type A2 (A_2A_R) and the release of the NMDAR coagonist d-serine. Specifically (1) the selective A_2A_R blocker 7-(2-phenylethyl)-5-amino-2-(2-furyl)-pyrazolo-[4,3-e]-1,2,4-triazolo[1,5-c]pyrimidine (SCH58261) and the genetic ablation of A_2A_R prevent CX_3_CL1 action while the A_2A_R agonist 5-(6-amino-2-(phenethylthio)-9H-purin-9-yl)-*N*-ethyl-3,4-dihydroxytetrahydrofuran-2-carboxamide (VT7) mimics CX_3_CL1 effect, and (2) the selective blocking of the NMDAR glycine (and d-serine) site by 5,7-dicholorokynurenic acid (DCKA), the enzymatic degradation of d-serine by d-amino acid oxidase (DAAO) and the saturation of the coagonist site by d-serine, all block the CX_3_CL1 effect. In addition, mass spectrometry analysis demonstrates that stimulation of microglia and astrocytes with CX_3_CL1 or VT7 increases d-serine release in the extracellular medium.

**Conclusions:**

CX_3_CL1 transiently potentiates NMDAR function though mechanisms involving A_2A_R activity and the release of d-serine.

## Introduction

It is now widely accepted that, in addition to their well established role in the immune system, chemokines and their receptors play important roles in the central nervous system (CNS), contributing to the homeostasis of mature brain through neuroendocrine modulation, neuromodulation and neuroglia communication (for review see [1] and [2]).

Fractalkine (CX_3_CL1) is one of the chemokines most abundantly expressed in the brain [3-5], especially by neurons, whereas the only known CX_3_CL1 receptor, CX_3_CR1, is expressed by microglial cells [5-7]. The CX_3_CR1/CX_3_CL1 axis plays a major role in neuron/microglia crosstalk and in neuroprotection under conditions of inflammation/injury [5,8-10]. We have previously shown that CX_3_CL1 reduces neuronal death induced by glutamate (Glu) [11] through microglia-derived protective factors including adenosine [12,13] and in rodent models of permanent ischemia, exogenous CX_3_CL1 administration is neuroprotective [14]. Moreover, at synapses between Schaffer collaterals and pyramidal neurons in the CA1 region of the hippocampus, CX_3_CL1 exerts a neuromodulatory role inducing α-amino-3-hydroxy-5-methyl-4-isoxazolepropionic acid receptor (AMPAR)-mediated excitatory synaptic depression and negatively modulating long-term potentiation (LTP) mediated by *N*-methyl-d-aspartate receptors (NMDAR) [15-17].

It is well established that NMDARs play an important role in a variety of physiological and pathological processes such as excitatory neurotransmission, synaptic plasticity and excitotoxicity [18,19]. NMDAR function is regulated by agents acting on a number of sites other than the Glu binding site [20], such as glycine [21] and d-serine that act with different affinities on the same binding site [22]. A recent report describes that d-serine preferentially acts on synaptic NMDARs, whereas glycine acts on extrasynaptic NMDARs [23]. In addition, d-serine is highly localized on glia in areas of the brain particularly enriched in NMDARs [24], thus suggesting that these cells are likely playing key roles in regulating NMDAR-dependent processes including synaptic transmission and synaptic plasticity. d-serine is degraded by d-amino acid oxidase (DAAO) and is mainly synthesized from l-serine by serine racemase (SR) present primarily on astrocytes. Recent studies also identified neurons and microglia as other important cellular sources of serine in the brain [25-29] even thought the role of neuronal and microglial d-serine remains to be determined.

Microglia have long been characterized by their innate immune activity in the nervous system, however recent evidence has shed new light on microglia function. These cells are now recognized as full time partners in neuronal function and the crosstalk between neurons and microglia has been described in several physiological and pathological conditions. Microglia secrete a number of factors, including Glu, nitric oxide, purines, trophic and anti-inflammatory substances [30], but also soluble factors affecting NMDAR and AMPAR such as glycine, cytokines, serine proteases and adenosine [31-34]. We have previously shown that stimulation of microglial cells with CX_3_CL1 induces the release of adenosine, which mediates the reduction of AMPAR function [13,15-17], but scarce information is available on specific CX_3_CL1 effects on hippocampal NMDARs [35] that are highly involved in synaptic plasticity processes.

In the present work we demonstrate that CX_3_CL1 modulates NMDA-mediated synaptic transmission in the hippocampal CA1 region, through the activity of the A_2A_R and the release of d-serine from glia.

## Methods

### Animals

Experiments were performed in agreement with international guidelines on the ethical use of animals from the European Communities Council Directive of 24 November 1986 (86/609 EEC). Hippocampal slices were routinely obtained from 1-month-old C57BL/6. When specified, hippocampal slices were obtained from gene modified animals: (1) CX_3_CR1^GFP/GFP^ mice on the C57BL/6 background (from Charles River, Calco, Italy), that were generated by replacing the first 390 bp of the *CX*_*3*_*CR1* gene with the *EGFP* gene [6,36]; (2) A_1_R KO mice [37] and (3) A_3_RKO mice [38] backcrossed at least ten times on a C57BL/6 background; (4) A_2_ knockout mice [39] on a BALB/cJ background (C;129S-*Adora2a*^*tm1Jfc*^/J) and control BALB/cJ wild-type mice (Charles River, Calco, Italy). C57BL/6 and BALB/cJ wild-type mice showed similar responses to CX_3_CL1 application (not shown).

### Hippocampal slice preparation

Briefly, animals were decapitated after being anesthetized with halothane. Whole brains were rapidly removed from the skull and immersed for 10 minutes in ice-cold artificial cerebrospinal fluid (ACSF) solution containing (1) for field recording experiments (in mM): NaCl 125, KCl 4.4, CaCl_2_ 2.5, MgSO_4_ 1.5, NaHPO_4_ 1, NaHCO_3_ 26 and glucose 10; (2) for patch clamp recordings (in mM): NaCl 125, KCl 2.5, NaH_2_PO_4_ 1.25, NaHCO_3_ 26, CaCl_2_ 2, MgCl_2_ 1, and glucose 10. The ACSF was continuously oxygenated with 95% O_2_, 5% CO_2_ to maintain the proper pH (7.4). Transverse (250 or 350 μm thick) slices were cut at 4°C with a vibratome (DSK, Japan) and the slices were placed in a chamber containing oxygenated ACSF. After their preparation, slices were allowed to recover for 1 h before recording.

For field recording experiments individual slices (350 μm thick) were then transferred to the interface slice-recording chamber (BSC1, Scientific System Design Inc., Mississauga, Ontario, Canada) with a total fluid dead space of approximately 3 ml. Slices were maintained at 30 to 32°C and constantly superfused at the rate of 2 ml/min. Solutions were applied to the slices by a peristaltic pump.

For patch-clamp experiments, individual slices (250 μm thick) were submerged in ACSF in the recording chamber at room temperature. The ACSF was perfused at a rate of approximately 1 ml/min.

In all experiments, unless otherwise specified, to isolate the NMDAR component, a modified ACSF (M-ACSF) containing low Mg^2+^ concentration (MgSO_4_ or MgCl_2_ were reduced to 0.2 mM) and the AMPA receptor blocker 2,3-dihydroxy-6-nitro-7-sulfamoyl-benzo[f]quinoxaline-2,3-dione (NBQX; 10 mM) were perfused for at least 20 minutes.

### Field potential recording

Slices were visualized with a Wild M3B microscope (Heerbrugg, Switzerland). At the beginning of each recording, a concentric bipolar stimulating electrode (SNE-100X 50 mm long Elektronik-Harvard Apparatus GmbH, Crisel Instruments, Rome Italy) was positioned in the stratum radiatum for stimulation of Schaffer collateral pathway projections to CA1. An ACSF-filled glass micropipette (0.5 to 1 MΩ) was positioned 200 to 600 μm from the stimulating electrode for recording the orthodromically-evoked NMDAR component of field excitatory postsynaptic potentials (NMDA-fEPSPs). Stimuli consisted of 100-μs-long constant current square pulses, applied at 0.05 Hz. The intensity of the stimulus was adjusted in each experiment to evoke approximately 50% of the maximal field potential amplitude without appreciable population spike contamination. Evoked responses were monitored online and stable baseline responses were recorded for at least 10 minutes. Only the slices that showed stable NMDA-fEPSP amplitudes were included in the experiments. To analyze the timecourse of the NMDA-fEPSPs slope, the recorded NMDA-fEPSPs were routinely averaged over 1 minute (N = 3). Experiments were performed in M-ACSF and averaged NMDA-fEPSPs (last 5 minutes of treatment or washout) were normalized to baseline values (5 minutes) prior to treatment. In the text, n refers to the number of slices analyzed/number of mice.

For the paired-pulse ratio (PPR) test, closely spaced consecutive stimuli (50 ms interval) were used, and PPR was calculated as the ratio between the NMDA-fEPSPs amplitude evoked by the second stimulus (A2) over the first (A1; A2/A1).

NMDA-fEPSPs were recorded and filtered (1 kHz) with an Axopatch 200 A amplifier (Axon Instruments, CA, USA) and digitized at 10 kHz with an A/D converter (Digidata 1322A, Axon Instruments). Data were stored on a computer using pClamp 9 software (Axon Instruments) and analyzed offline with the Clampfit 9 program (Axon Instruments).

### Patch-clamp recordings

Neurons were visualized at 640 × with Nomarski optics with an upright Zeiss (Thornwood, NY, USA) Axioscope microscope. Patch-clamp recordings were obtained using glass electrodes (3 to 4 MΩ) filled with the following (in mM): CsMeSO_4_ 135, CsCl 4, MgCl_2_ 2, 2-[4-(2-hydroxyethyl)piperazin-1-yl]ethanesulfonic acid (HEPES) 10, MgATP 2, NaGTP 0.3, (1,2-bis(o-aminophenoxy)ethane-*N*,*N*,*N*',*N*'-tetraacetic acid (BAPTA) 5 (pH 7.3, with CsOH). Neurons were clamped at −70 mV and perfused with M-ACSF. NMDA currents in standard ACSF were recorded at positive potential to remove Mg block (10 mV) using the following solution (in mM): CsCl 130, MgCl_2_ 2, HEPES 10, MgATP 2, NaGTP 0.3, BAPTA 5 (pH 7.3, with CsOH).

Membrane currents, recorded with a patch-clamp amplifier (Axopatch 200A; Molecular Devices, Foster City, CA, USA), were filtered at 2 kHz, digitized (10 kHz), and acquired with pClamp 9 software (Molecular Devices, Sunnyvale CA, USA). Excitatory postsynaptic currents (EPSCs) were evoked in CA1 pyramidal neurons by electrical stimulation with theta glass tubes pulled to a final tip diameter of 10 to 20 μm and filled with external solution. Stimulating electrodes were placed in the stratum radiatum to activate the Schaffer collateral pathway projecting to CA1. NMDA-EPSCs were evoked by stimulating at 5 to 50 V for 150 to 300 μs every 20 s. Recorded EPSCs were routinely averaged over 1 minute (N = 3) to analyze the timecourse of EPSC amplitude. Averaged NMDA-EPSCs (last 5 minutes of treatment or washout) were normalized to baseline values (5 minutes) prior to treatment. Stable EPSCs were monitored for at least 10 minutes before applying CX_3_CL1 and successively monitored during slice exposure to CX_3_CL1 (5 nM, a concentration close to the IC50 value for synaptic depression of AMPA mediated responses and to the concentration that inhibits NMDA-dependent LTP [15-17]. Data were analyzed offline with Clampfit 9 (Axon Instruments).

### Glia and neuron primary cultures

Primary cortical glial cells were prepared from 0 to 2-day-old mice. Cerebral cortices were chopped and digested in 30 U/ml papain for 40 minutes at 37°C followed by gentle trituration. The dissociated cells were washed, suspended in Dulbecco’s modified Eagle medium (DMEM) with 10% fetal bovine serum (FBS) (Invitrogen, Life Technologies, Monza, Italy) and 2 mM l-glutamine and plated at a density of 9 to 10 × 10^5^ in 175 cm^2^ cell culture flasks. At confluence (11 days *in vitro*), glial cells were plated (5.0 × 10^5^ cells/cm^2^) on dishes coated with poly-l-lysine (100 μg/ml) in DMEM supplemented with 10% FBS, 100 U/ml penicillin, 0.1 mg/ml streptomycin. To detach and collect microglial cells, glial cells at confluence (11 days *in vitro*) were shaken for 2 h at 37°C. These procedures gave almost pure microglia (<2% astrocyte contamination) and astrocytes cell population (4% to 6% of microglia contamination), as verified by staining for glial fibrillary acidic protein (GFAP) and isolectin IB_4_.

Cortical neuronal cultures were prepared from newborn C57BL/6 mice (P0 to P1). Cerebral cortex were chopped and digested in 20 U/ml papain for 40 minutes at 37°C. Cells (13 × 10^4^ cells/cm^2^) were plated on dishes coated with poly-l-lysine (100 μg/mL) in basal medium Eagle (BME) supplemented with 1 mM sodium pyruvate, 30 mM glucose, 0.1% Mito™ serum extender, 10% FBS, 100 U/mL penicillin, 0.1 mg/mL streptomycin and 10 mM HEPES-NaOH (pH 7.4). After 4 h the medium was changed with Neurobasal medium supplemented with 1 mM glutamine, 0.1% Mito™ serum extender, 2.5% B27, 100 U/ml penicillin and 0.1 mg/mL streptomycin. After 2 days, AraC (5 μM) was added to avoid the growth of glial cells. The percentage of neuronal cells obtained is 80% to 90%, as determined with β-tubulin III staining. Cultures were used after 10 to 11 days.

### Measurement of d-serine by liquid chromatography and mass spectrometry (MS) analysis

Mixed glia, microglia and neuronal cultures at 11 days old were pretreated in M-ACSF for 40 minutes and stimulated for either 20 minutes, 2 h and 20 minutes and 4 h with CX_3_CL1 10 nM or vehicle. After this time, media were collected, centrifuged at 4°C for 10 minutes at high speed*,* and the resulting supernatants were kept to −20°C until use. Cells remaining in the dish were analyzed for protein content with a BCA assay (Pierce, Euroclone Pero, Italy).

Aliquots of the culture medium was derivatized according to the method described by Berna and Ackermann [40]. Briefly, samples (25 μl) were incubated with 200 mM (pH 7.5) sodium bicarbonate solution and 10 μl of 1% Marfey’s reagent (w/v in acetone) at 60°C for 1h.

Next, the samples were evaporated to dryness at 60°C under a stream of nitrogen, and the dried residues were reconstituted with 12.5% methanol in 15 mM ammonium acetate. The samples were centrifuged at 600 *g* for 5 minutes and the supernatants diluted with 200 μl of mobile phase. Injections of 20 μl were performed for liquid chromatography-tandem mass spectrometry (LC-MS/MS) analysis. Marfey’s adducts of D standard mixture were prepared by appropriate dilution of the 100 μg/ml stock solution of d-serine in high-performance liquid chromatography (HPLC)-grade water and following the same derivatization procedure.

The LC-MS/MS system consisted of a PerkinElmer 200 Series binary pump and autosampler (PerkinElmer, Norwalk, CT, USA) and an SCIEX API2000MS/MS triple quadrupole mass spectrometer including a Turbolon Spray® ionization source (Applied Biosystem-MDS SCIEX, Thornhill, Ontario, Canada).

Chromatographic separation was performed on a Synergi Polar RP column (150 × 2.0 mm, 4 μm), protected by a guard column with identical packing material (4 × 2.0 mm; Phenomenex, Torrance, CA, USA) The mobile phase consisted of a linear gradient (30% to 70% with respect to acetonitrile) formed by combination of 5 mM ammonium formate buffer in water (pH 4.0, eluent A) and acetonitrile (eluent B) at a flow rate of 0.2 ml/min.

d-Serine derivatives were detected using multiple reaction monitoring (MRM) in negative ionization mode. Selected ion masses of the protonated precursors and fragmented ions (*m*/*z*) were 359.1 > 192. Chromatographic peaks were integrated using Analyst™ software (version 1.4.1, SCIEX). The detection limits (LOD) and quantification limits (LOQ) for d-serine were 1.71 ng/ml and 5.18 ng/ml respectively. The Marfey’s derivated isobaric compounds l-Ser and d-Ser (2,4-dinitro-phenyl-5-l-alanine amide- (DNPA-)l-Ser and DNPA-d-Ser) were well resolved chromatographically as shown in Additional file 1: Figure S1. An example of the blank samples (control M-ACSF) is shown in Additional file 2: Figure S2, where no signal corresponding to DNPA-l-Ser or DNPA-d-Ser are present.

### Statistical analysis

The values were reported as mean ± SEM. Unless otherwise specified, data values refer to number of slices/number of animals analyzed. Where appropriate, t tests or one-way analysis of variance (ANOVA) were used. Since in all experiments data passed the normality and the equal variance tests, we performed parametric ANOVA followed by Tukey or Holm-Sidak multiple-comparison test (Sigma Plot, 11.0 software). If not specified, *P* values refer to ANOVA *post hoc* analyses.

### Drugs

CX_3_CL1 (human, Peprotech Inc. Rocky Hill, NJ, USA), 7-(2-phenylethyl)-5-amino-2-(2-furyl)-pyrazolo-[4,3-e]-1,2,4-triazolo[1,5-c]pyrimidine (SCH58261, stock solution 10 mM in dimethylsulfoxide (DMSO)), triazoloquinazoline (CGS15943, stock solution 5 mM in DMSO) and 1,3-dipropyl-8-cyclopentylxanthine (DPCPX stock solution 5 mM in DMSO) by Tocris Bioscience, Bristol, UK. 3-Propyl-6-ethyl-5-[(ethylthio)carbonyl]-2 phenyl-4-propyl-3-pyridine carboxylate (MRS1523, stock solution 10 mM), catalase, d-amino acid oxidase (DAAO), Marfey’s reagent (Nα-(2,4-dinitro-5-fluophenyl)-l-alaninamide), ammonium formate and Minocycline (stock solution 20 mM) from Sigma-Aldrich (Milan, Italy). NBQX (stock solution 20 mM), 5,7-dicholorokynurenic acid (DCKA, stock solution 750 μM in 1 eq. NaOH) and d-serine (stock solution 10 mM) by Ascent Scientific, Bristol, UK. 5-(6-Amino-2-(phenethylthio)-9H-purin-9-yl)-*N*-ethyl-3,4-dihydroxytetrahydrofuran-2-carboxamide (VT7, stock solution 10 mM in DMSO). Adenosine deaminase (ADA, Roche, Germany). Acetonitrile, methanol, both gradient grades were purchased from Merck (Darmstadt, Germany). Ultrapure water was prepared using a Millu-Q system (Millipore, MA, USA).

Drugs were dissolved in ACSF just before application.

## Results

### CX_3_CL1 increases hippocampal NMDA responses

**Figure 1:**
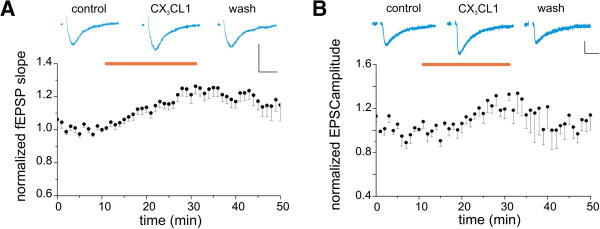
**CX**_**3**_**CL1 produces transient *N*-methyl-d-aspartate receptor (NMDAR) potentiation in CA1 pyramidal neurons (STP).** Horizontal bar, CX_3_CL1 application (5 nM). **(A)** CX_3_CL1 increases NMDA-field excitatory postsynaptic potentials (fEPSPs) slope. Top: representative average traces of NMDA-fEPSPs recorded in control (1 minute before application), after 20 minutes of CX_3_CL1 treatment and at 20 minutes of withdrawal, as indicated. Vertical scale bar: 0.2 mV, horizontal scale bar: 20 ms. Bottom: average timecourse of CX_3_CL1-induced increase of the NMDA-fEPSPs slope in mouse hippocampal slices (n = 35/28). Here and thereafter each point represents the average of three responses evoked every 20 s, as detailed in Methods. **(B)** CX_3_CL1 increases the amplitude of evoked excitatory postsynaptic currents (EPSCs). Top: sample traces of EPSCs (average of three events over 1 minute) recorded before, after 20 minutes of chemokine application, and 20 minutes after washout, as indicated. Vertical scale bar: 20 pA, horizontal scale bar: 20 ms. Bottom: average timecourse of the current peak amplitude. Circles represent averages of three responses evoked every 20 s in five different slices (five mice).

To investigate whether CX_3_CL1 influences NMDA-mediated synaptic transmission in hippocampal CA1 region, we recorded the NMDAR component of the fEPSPs (NMDA-fEPSPs) elicited in the CA1 stratum radiatum of mouse hippocampal slices by electrical stimulation of the Schaffer collateral (at 0.05 Hz). To isolate the NMDA component of the fEPSPs slices were treated with the AMPA receptor blocker NBQX (10 μM) and with low (0.2 mM) magnesium [41]. In control slices, synaptic activity was stable for at least 30 minutes (not shown). We challenged the slices with CX_3_CL1 at concentrations of 5 nM. In the majority of the experiments (25 out of 35 slices tested, 28 mice; Figure 1A) bath application of CX_3_CL1 for 20 minutes induced a short-term potentiation (STP) of synaptic transmission. The potentiation of the NMDA-fEPSPs slope developed within 5 minutes of CX_3_CL1 application was 1.22 ± 0.03 of baseline (from 1.92 to 1.01 in different experiments, measured here and thereafter after 20 minutes of treatment, *P* <0.05) and was followed by partial recovery (to 1.15 ± 0.01 of baseline, *P* <0.05, measured here and thereafter at 20 minutes of CX_3_CL1 withdrawal) with a few exceptions where full recovery was observed. To exclude the possibility that CX_3_CL1 treatment induces a change in presynaptic cell excitability, such as the recruitment of more presynaptic fibers by the same stimulus, changes in the amplitude of extracellular fiber volley (an index of presynaptic excitation) were monitored by recording evoked fEPSPs during CX_3_CL1 application. As shown in Additional file 3: Figure S3A, in a subset of slices where the potentiating effect of CX_3_CL1 was maximal (41.68 ± 0.05 of baseline, n = 5, *P* <0.05) the amplitude of the afferent volley did not change after treatment with CX_3_CL1 (*P* = 0.45, paired *t* test).

Patch-clamp experiments were carried out to measure CX_3_CL1-mediated modulation of NMDA current at the single cell level. Whole-cell recordings of NMDA-EPSCs were performed in CA1 pyramidal neurons in acute slices while repeatedly stimulating Schaffer collateral axons. CX_3_CL1 treatment caused an increase in NMDA-EPSC peak amplitude (to 1.25 ± 0.03 *P* < 0.05 vs baseline) that recovered upon withdrawal (to 1.06 ± 0.04 *P* >0.05 vs baseline, Figure 1B, five slices/five mice). These results confirmed the CX_3_CL1-mediated potentiation of NMDA responses also at the single cell level.

### Paired pulse facilitation (PPF) ratio is not altered by CX_3_CL1 application

To analyze if CX_3_CL1 potentiates NMDA-fEPSPs by altering presynaptic glutamate release, we performed studies of short-term plasticity, that is, paired-pulse ratio (PPR) measurements. The PPR, which is the ratio of the amplitude of the second response vs the first, depends on the probability of vesicular release at synapse and its variation is generally associated with changes in transmitter release probability [42].

To evaluate PPR values we stimulated Schaffer collateral pathway projections to CA1 at 50 ms intervals. As shown in Additional file 3: Figure S3B, PPR was 1.53 ± 0.017 and 1.54 ± 0.026 before and after 20 min of CX_3_CL1 treatment (6 slices/4 mice, *P* = 0.74, paired *t* test), respectively.

Presynaptic release of Glu can be affected by removal of Mg^2+^ from the external solution, changing the competition between Mg^2+^ and Ca^2+^ at terminal endings and altering PPR mechanisms [43]. To investigate this possibility we repeated the PPR measurements in whole-cell experiments where the NMDA current were isolated by depolarizing the cell in a standard ACSF medium. As shown in Additional file 3: Figure S3C, the PPR measured in this condition was 1.43 ± 0.08 and 1.44 ± 0.09 before and after 20 minutes of CX_3_CL1 treatment, (5 slices/5 mice, *P* = 0.81, paired *t* test). These data indicated that under our experimental conditions, CX_3_CL1 treatment did not cause obvious changes in presynaptic release probability, confirming the postsynaptic nature of the potentiation of NMDA-fEPSPs, in line with our previous findings [15,44].

### CX_3_CL1-mediated effects require the presence of CX_3_CR1 on microglial cells

**Figure 2:**
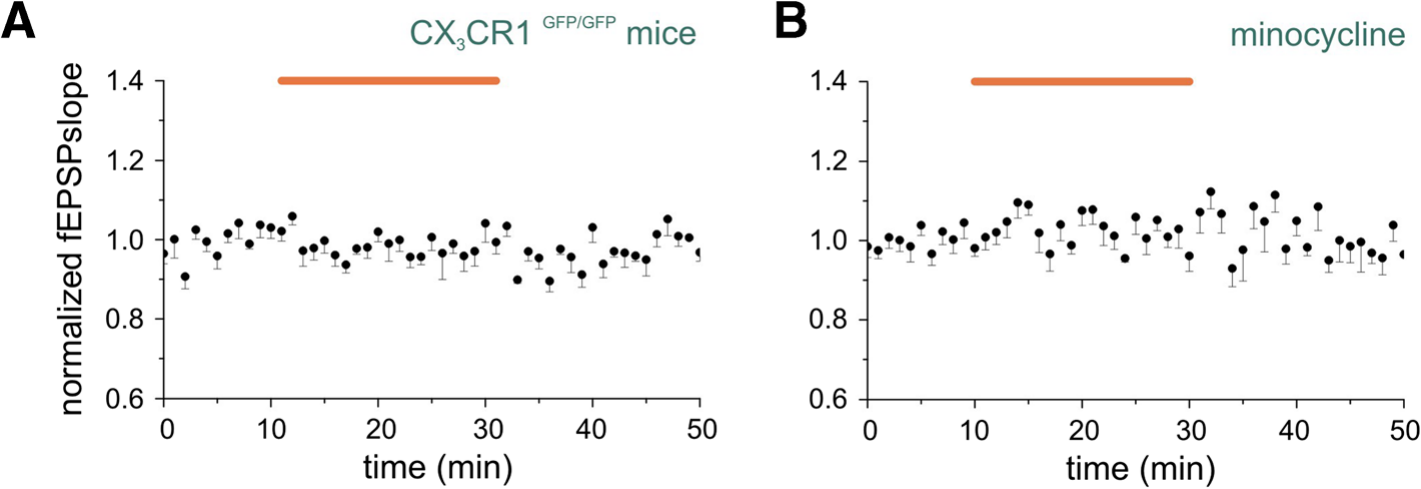
**CX**_**3**_**CR1 and microglia activation are required for CX**_**3**_**CL1 effects.** Points represent mean ± SEM of the *N*-methyl-d-aspartate receptor component of field excitatory postsynaptic potential (NMDA-fEPSP) slopes. Average timecourse of changes in NMDA-fEPSPs recorded in hippocampal slices. Horizontal bar, CX_3_CL1 application (5 nM). **(A)** CX_3_CL1-mediated effects required the presence of CX_3_CR1 (n = 7/3). **(B)** Microglia activation is necessary for CX_3_CL1 action. Slices pretreated for 1 h with minocycline (last 10 minutes in the graph) and then continuously superperfused. Cotreatment with CX_3_CL1 did not increase NMDA-fEPSPs (n = 8/2).

To demonstrate that the effects of CX_3_CL1 were indeed mediated by CX_3_CR1, experiments were performed on CX_3_CR1^GFP/GFP^ mice where the murine CX_3_CR1 gene was replaced with the cDNA encoding EGFP (Jung *et al*. [6]). Application of CX_3_CL1 for 20 minutes to CX_3_CR1^GFP/GFP^ hippocampal slices (Figure 2A, seven slices/three mice) failed to induce any obvious basal NMDA-fEPSPs slope change (*P* > 0.05). These findings clearly show that CX_3_CL1-induced effects on NMDA-fEPSPs were mediated through CX_3_CR1 activation.

Since CX_3_CR1 is expressed on microglial cells [5,6], we wondered whether microglial activation was required for CX_3_CL1-mediated STP of NMDA-fEPSPs. For this purpose we treated hippocampal slices with a broad-spectrum tetracycline antibiotic, minocycline, known to inhibits microglial activation [45-47].

As shown in Figure 2B, in the presence of minocycline (20 μM, 1 h preincubation), the application of CX_3_CL1 did not significantly affect the NMDA-fEPSPs (*P* > 0.5, eight slices/two mice), demonstrating a microglia-driven chemokine effect.

### CX_3_CL1-mediated NMDAR potentiation is mediated by ARs

**Figure 3:**
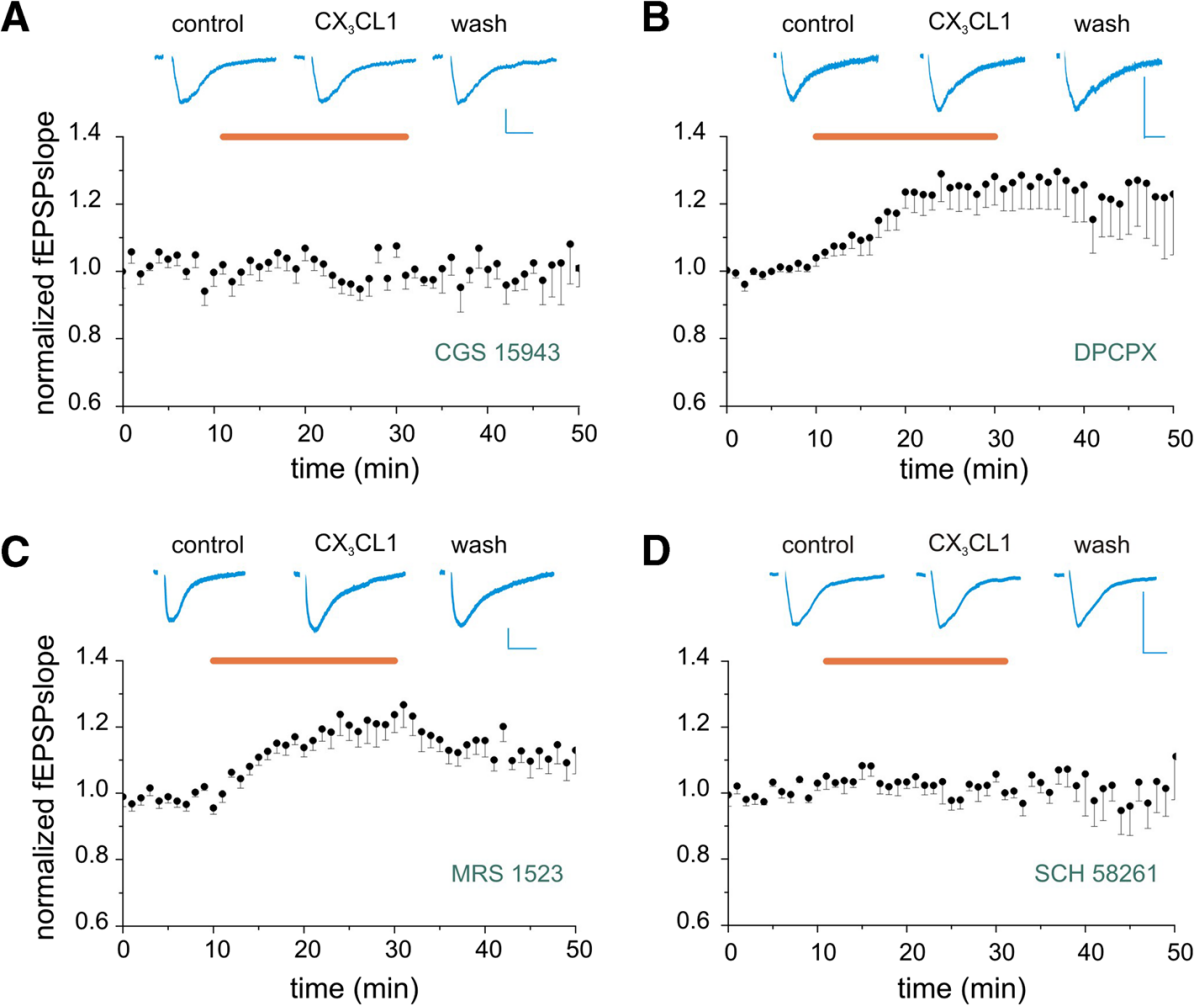
**Adenosine receptors (ARs) are involved in CX**_**3**_**CL1-mediated short-term potentiation (STP) of *N*-methyl-d-aspartate receptor (NMDAR). (A-D)** Slices pretreated for 20 minutes with AR antagonists (last 10 minutes in the graph) and then continuously treated. Inset on top: representative NMDAR component of field excitatory postsynaptic potential (NMDA-fEPSP) traces recorded in control condition, in the presence of CX_3_CL1 and during washout, as indicated (same time points as Figure 1). Vertical scale bar: 0.5 mV, horizontal scale bar: 20 ms. Graphs represent the average timecourse of CX_3_CL1 effects on NMDA-fEPSPs in the presence of AR antagonists. Points: mean ± SEM. Horizontal bar, CX_3_CL1 application (5 nM). (A) Slices superfusion with the non-selective AR antagonist, triazoloquinazoline (CGS15943) (25 nM) prevented CX_3_CL1 action (n = 9/4). **(B)** CX_3_CL1 application in the presence of 1,3-dipropyl-8-cyclopentylxanthine (DPCPX) (25 nM), was effective (n = 9/5). (C) CX_3_CL1 application in the presence of 3-propyl-6-ethyl-5-[(ethylthio)carbonyl]-2 phenyl-4-propyl-3-pyridine carboxylate (MRS1523) (100 nM) induced a significant increase of NMDA-fEPSPs (n = 10/5). (D) 7-(2-phenylethyl)-5-amino-2-(2-furyl)-pyrazolo-[4,3-e]-1,2,4-triazolo[1,5-c]pyrimidine (SCH58261) (10 nM) prevented CX_3_CL1 action (n = 6/3).

We have previously shown that CX_3_CL1 treatment induces the release of adenosine from microglia [12] and that CX_3_CL1-mediated modulation of synaptic transmission and plasticity involves AR activity [17,48]. In addition, it is known that AR activity in the hippocampus modulates NMDA receptor functioning [49]. For all these reasons we investigated whether CX_3_CL1 effects on NMDARs require AR activity. We first treated hippocampal slices with the broad-spectrum AR antagonist CGS15943. The applications of CGS15943 (1 μM) abolished CX_3_CL1-mediated STP of NMDA-fEPSPs (Figure 3A, *P* > 0.05 vs baseline, nine slices/four mice) indicating that the activity of ARs is indeed involved in CX_3_CL1 effects.

To disclose the specific role of distinct AR subtypes, we performed additional experiments with selective A_1_R, A_2A_R and A_3_R antagonists (Figure 3B-D).

Specifically, the A_1_R antagonist DPCPX (25 nM) failed to prevent CX_3_CL1-mediated modulation of NMDAR, as the NMDA-fEPSPs slope increased during CX_3_CL1 treatment (to 1.25 ± 0.01, *P* < 0.05 vs baseline, *P* > 0.05 vs CX_3_CL1 alone) and remained high upon withdrawal (to 1.25 ± 0.04 *P* < 0.05 vs baseline, Figure 3B, nine slices/five mice). Similarly, the specific A_3_R antagonist MRS1523 (100 nM) was unable to affect the action of CX_3_CL1 that elicited a significant increase in NMDA-fEPSPs slope (to 1.23 ± 0.01, *P* < 0.05 vs baseline, *P* > 0.05 vs CX_3_CL1 alone) partially recovering upon withdrawal (1.12 ± 0.01, *P* < 0.05 vs baseline, Figure 3C, ten slices/five mice).

Conversely, when slices were treated with the selective A_2A_R antagonist SCH58261 (10 nM), CX_3_CL1 was unable to affect NMDA-fEPSPs slope (Figure 3D, *P* > 0.05 vs baseline *P* < 0.01 vs CX_3_CL1 alone, six slices/three mice), indicating that A_2A_R activity is associated with CX_3_CL1 effects on NMDAR-dependent basal transmission.

**Figure 4:**
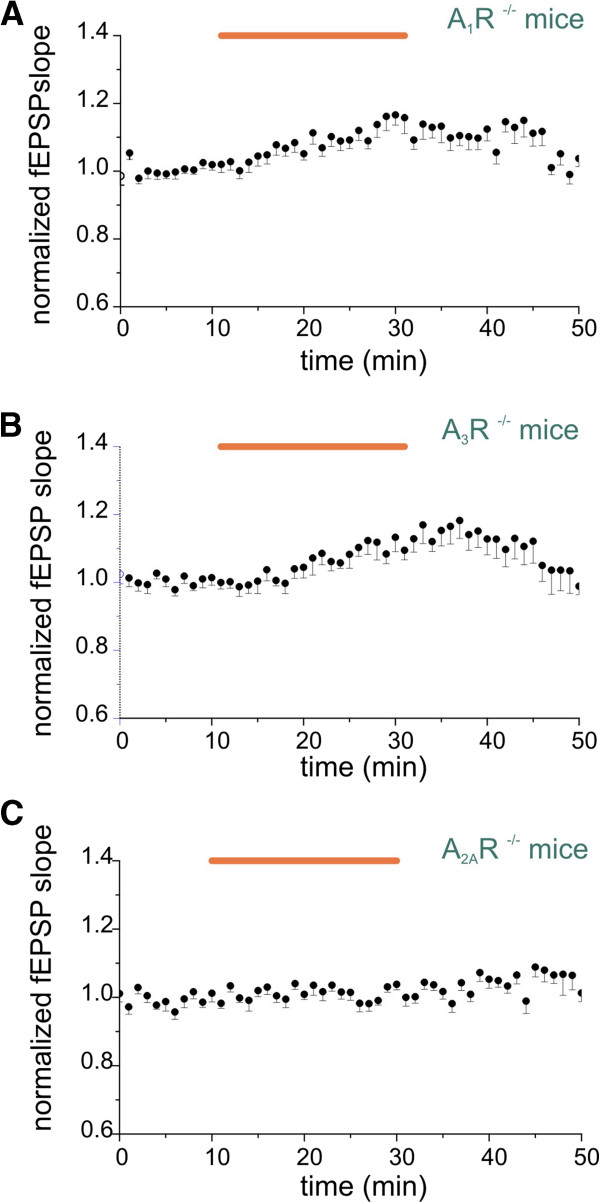
**The absence of adenosine receptor type A2 (A**_**2A**_**R) prevents CX**_**3**_**CL1 action.** Graphs represent the average timecourse of CX_3_CL1 effects on *N*-methyl-d-aspartate receptor component of field excitatory postsynaptic potentials (NMDA-fEPSPs). Points: mean ± SEM. Horizontal bar, CX_3_CL1 application (5 nM). **(A)** In A_1_R^−/−^ mice, application of CX_3_CL1 enhanced NMDA-fEPSPs (n = 10/3). **(B)** The absence of A_3_R did not interfere with CX_3_CL1 action (n = 10/2). **(C)** In A_2A_R^−/−^ mice, CX_3_CL1 was ineffective (n = 11/3).

**Figure 5:**
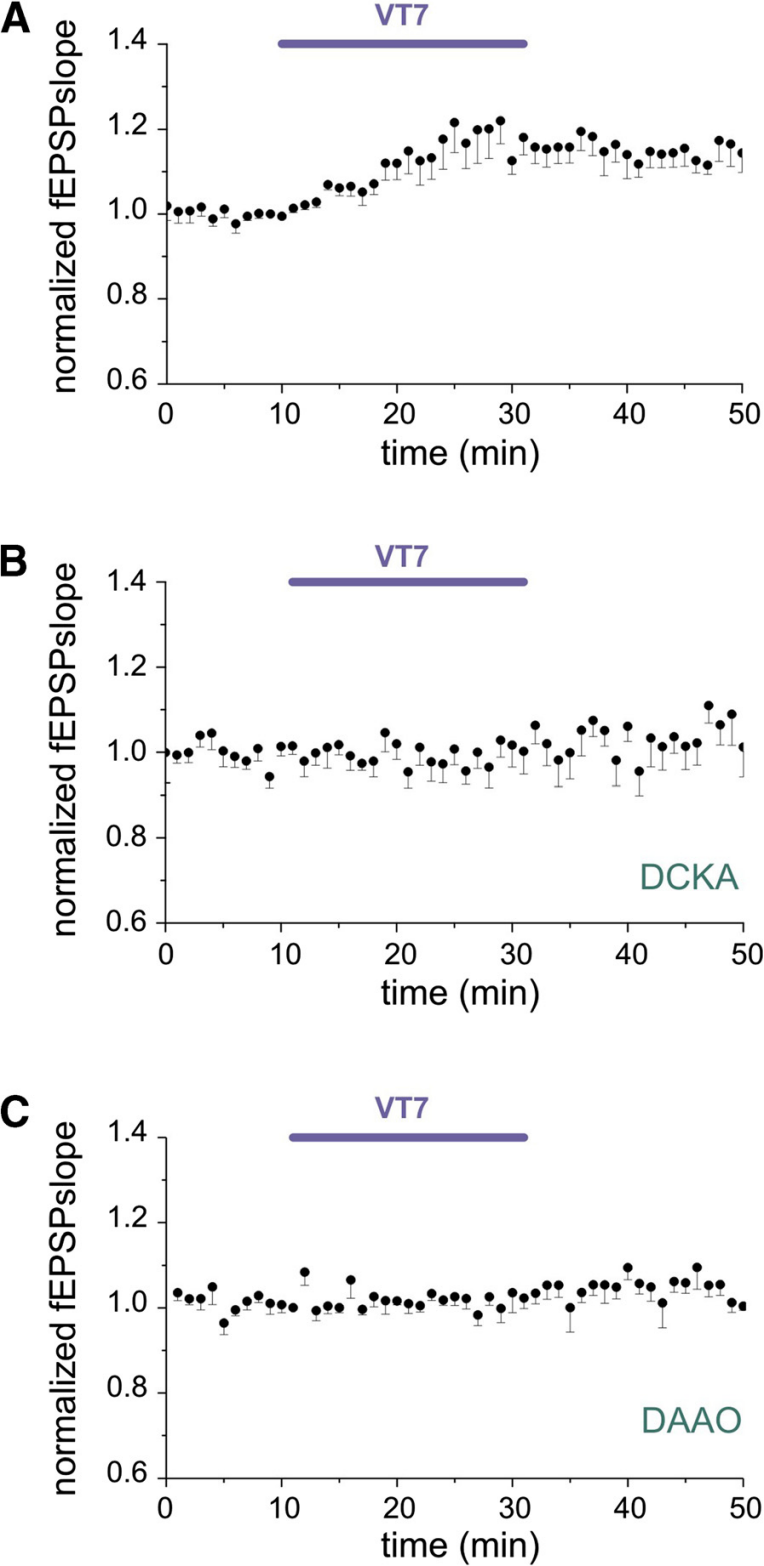
**5-(6-Amino-2-(phenethylthio)-9H-purin-9-yl)-*N*-ethyl-3,4-dihydroxytetrahydrofuran-2-carboxamide (VT7) increases hippocampal*N*-methyl-d-aspartate receptor component of field excitatory postsynaptic potentials (NMDA-fEPSPs) and its action depends on d-serine.** Averaged timecourse of changes in NMDA-fEPSPs recorded in hippocampal slices. Points: mean ± SEM. Horizontal bar, VT7 application (50 nM). Inset on top: representative NMDA-fEPSP traces recorded in control conditions, in the presence of VT7 and during washout, as indicated (same time points as Figure 1). Vertical scale bar: 0.1 mV, horizontal scale bar: 20 ms. Slices treated with adenosine deaminase (ADA), 1 U/ml. **(A)** Application of VT7 enhances NMDA-fEPSP slopes (n = 9/2). **(B)** Blocking of the NMDAR d-serine site completely prevents the VT7 effect on NMDA-fEPSP slopes. Slices pretreated with 5,7-dicholorokynurenic acid (DCKA) (750 nM) for 20 minutes (last 10 minutes in the graph) before VT7 application and then continuously treated (n = 11/3). **(C)** Degradation of d-serine block VT7-mediated NMDA modulation. Slices pretreated for 1 h and then continuously superperfused with d-amino acid oxidase (DAAO) (0.1 U/ml) and catalase (300 U/ml). Cotreatment with VT7 did not increase NMDA-fEPSPs slope (n = 7/3).

The role of ARs in CX_3_CL1 effects was further investigated by means of mice selectively lacking AR subtypes. As shown in Figure 4A application of CX_3_CL1 in A_1_R^−/−^ mice increased NMDA-fEPSPs slope (to 1.14 ± 0.01, *P* < 0.05 vs baseline) that partially recovered after washout (to 1.04 ±0.02, *P* > 0.05 vs baseline, ten slices/three mice). Similar results were obtained in A_3_R^−/−^ mice, where CX_3_CL1 elicited a significant increase of NMDA-fEPSPs slope (to 1.11 ± 0.01, *P* < 0.05 vs baseline) that recovered upon withdrawal (to 1.02 ± 0.01, *P* > 0.05 to baseline, Figure 4B, ten slices/two mice). Interestingly, in accordance with pharmacological data, in A_2A_R deficient mice (A_2A_R^−/−^) CX_3_CL1 was unable to affect NMDA-fEPSPs. (*P* > 0.05, vs baseline, 11 slices/3 mice, Figure 4C), thus pointing to an essential role of A_2A_R activity in the CX_3_CL1 effect. To further support this observation, we tested whether a selective A_2A_R agonist, VT7 (50 nM), was able to mimic CX_3_CL1 effect. The results in Figure 5A demonstrate that in the presence of adenosine deaminase (ADA, 1 U/ml), which removes ambient adenosine [50], VT7 induced an increase of NMDA-fEPSPs slope (to 1.18 ± 0.01, *P* < 0.05 vs baseline, nine slices/two mice) that did not recover after washout (to 1.15 ± 0.01, *P* < 0.05 vs baseline).

Considered together, our findings indicate that the influence of CX_3_CL1 on NMDARs is mediated by the A_2A_R activity.

### d-serine is involved in CX_3_CL1-dependent modulation of NMDARs

Recent evidence indicates that d-serine serves as indispensable cofactor at the glycine binding site of NMDARs representing a new and important glia-derived neuromodulatory factor [51]. In fact, in glial cells, d-serine is synthesized and metabolized endogenously by the enzymes serine racemase (SR) [52] and d-amino acid oxidase (DAAO) [53], respectively.

**Figure 6:**
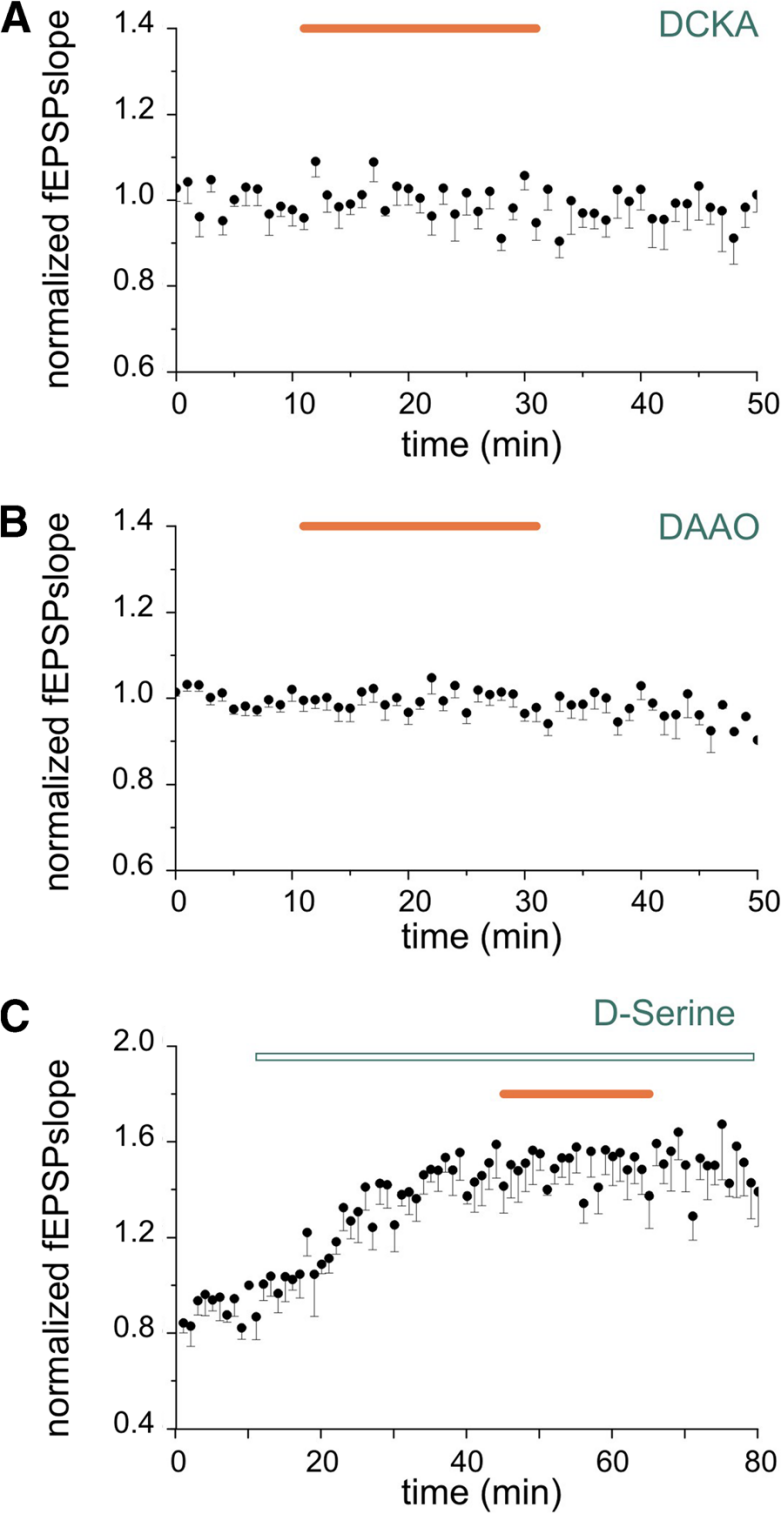
**d-Serine is involved in CX**_**3**_**CL1 action.** Graphs represent the average timecourse of *N*-methyl-d-aspartate receptor component of field excitatory postsynaptic potentials (NMDA-fEPSPs). Points: mean ± SEM. Black horizontal bar, CX_3_CL1 application (5 nM). **(A)** Blocking of NMDAR glycine site prevented CX_3_CL1 effects. Slices pretreated with 5,7-dicholorokynurenic acid (DCKA) (750 nM) for 20 minutes before CX_3_CL1 applications (last 10 minutes in the graph) and then continuously treated. Note the absence of CX_3_CL1 effect (n = 8/4). **(B)** Enzymatic degradation of d-serine abolished CX_3_CL1-mediated NMDAR modulation. Slices pretreated for 1 h and then continuously superperfused with d-amino acid oxidase (DAAO) (0.1 U/ml) and catalase (300 U/ml). Cotreatment with CX_3_CL1 did not increase NMDA-fEPSP slopes (n = 10/4). **(C)** Saturation of NMDAR glycine site by d-serine prevented CX_3_CL1 action. Treatment with d-serine (10 μM, open bar as indicated) increased basal NMDA-fEPSPs slope and occluded CX_3_CL1 effect (n = 6/4).

To investigate whether d-serine is involved in CX_3_CL1-mediated modulation of NMDARs, we first treated the slices with the selective NMDAR glycine-site antagonist 5,7-dichlorokynurenic acid (DCKA) at a concentration (750 nM) known to reduce NMDA responses by around 25% [54] and able to prevent d-serine mediated increase of NMDA-fEPSPs slope (n = 7, data not shown). At this concentration, DCKA abolished CX_3_CL1 effects on NMDA-fEPSPs (*P* > 0.05 vs baseline, eight slices/four mice, Figure 6A).

To disclose a possible d-serine versus glycine coagonist requirement, we treated the slices with DAAO (0.1 U/ml). Excess catalase (300 U/ml) was added in the solution in order to avoid the possibility of hydrogen peroxide production from the enzymatic degradation of d-serine by DAAO. Under this condition, DAAO completely prevented CX_3_CL1 action (*P* > 0.05 vs baseline, ten slices/four mice, Figure 6B), indicating that d-serine is necessary. To further confirm the involvement of d-serine in the modulation of NMDARs by CX_3_CL1, we applied exogenous d-serine (10 μM, six slices/four mice, Figure 6C) to hippocampal slices. As shown in Figure 6C, d-serine produced an increase in NMDA-fEPSPs slope reaching a plateaux effect within 20 minutes (up to 64.4 ± 0.01 after 40 minutes treatment, *P* < 0.05 vs baseline). Subsequent coapplication of CX_3_CL1 failed to further increase NMDA-fEPSPs slope (*P* > 0.05 vs d-serine alone), indicating that saturation of the glycine site by d-serine interferes with chemokine action.

Since we have demonstrated that the CX_3_CL1 effect is prevented by hampering both d-serine and A_2A_R functions, we investigated whether the two pathways share some common mechanisms of action. For this reason we applied the A_2A_R agonist VT7 (in the presence of ADA, 1 U/ml) while partially blocking the NMDAR d-serine site by DCKA. In this condition, VT7 failed to increase NMDA-fEPSPs slope (*P* > 0.05 vs baseline, Figure 5B, 11 slices/3 mice). In addition, slices pretreatment with DAAO (0.1 U/ml, in the presence of catalase, 300 U/ml, and ADA, 1 U/ml) inhibits VT7 action (>0.05 vs baseline, Figure 5C, seven slices/three mice). Taken together this evidence suggests that NMDAR modulation mediated by A_2A_R requires d-serine.

### CX_3_CL1 and VT7 stimulate the release of d-serine from glia cells

To further investigate the possible source of d-serine in CX_3_CL1-induced NMDAR modulation, we measured d-serine levels in glia-conditioned medium. The control medium (M-ACSF) did not contain d-serine, as it is shown in Additional file 2: Figure S2.

Primary cortical glial cells and isolated microglial cell populations were stimulated with CX_3_CL1 (10 nM) for different times (20 minutes, 2 h and 20 minutes or 4 h). After stimulation, mass spectrometry was performed on media samples.

**Figure 7:**
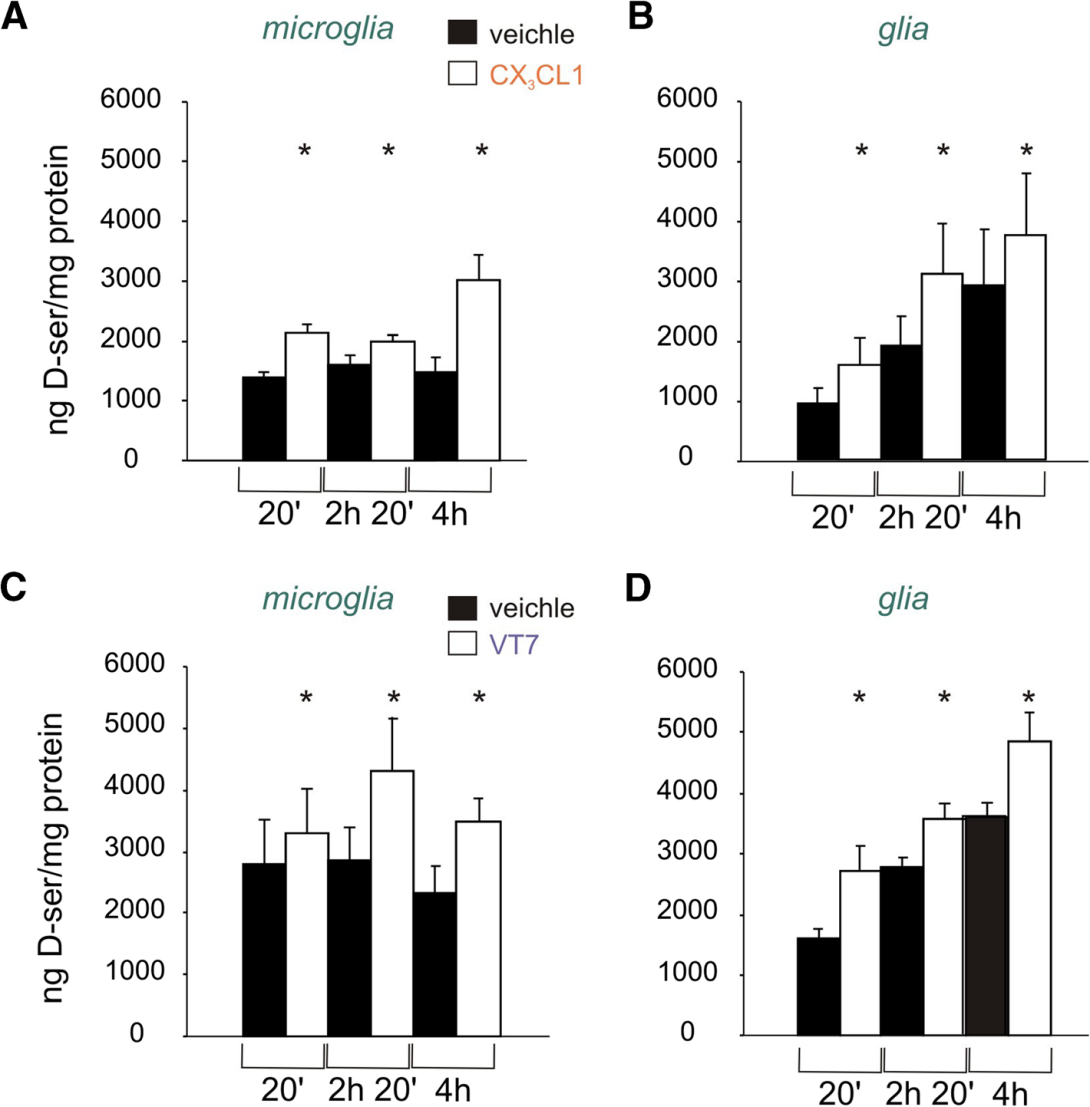
**CX**_**3**_**CL1 and 5-(6-amino-2-(phenethylthio)-9H-purin-9-yl)-*N*-ethyl-3,4-dihydroxytetrahydrofuran-2-carboxamide (VT7) induce extracellular accumulation ofd-serine.** Mass spectroscopy analysis of d-serine levels in primary purified microglia or mixed glia culture medium **(A,B)**. Cells were stimulated with CX_3_CL1 (10 nM) or vehicle and after 20 minutes, 2 h and 20 minutes or 4 h (as indicated) medium was collected and analyzed for d-serine content. Results are expressed as ng extracellular d-serine accumulated per mg of cellular proteins and are the mean ± SEM of eight independent experiments. **(A)** Shows purified microglia, **(B)** shows mixed glia culture medium. **(C,D)** Cells stimulated with VT7 (50 nM) plus adenosine deaminase (ADA, 1 U/ml) or vehicle (ADA, 1 U/ml) and samples collected as in (A,B). **(C)** Shows purified microglia, **(D)** shows mixed glia culture medium. Results are the mean ± SEM of nine experiments **(C)** or 12 experiments **(D)**. White square, vehicle; black square treatment, as indicated. *P <0.05.

Treatment of both primary purified microglia and mixed glia with CX_3_CL1 resulted in a large increase in d-serine in the medium (Figure 7). In particular, in microglial cells CX_3_CL1 induced a significant increase in d-serine release at all the incubation times considered (1.53 ± 0.08 after 20 minutes, 1.26 ± 0.07 after 2 h and 20 minutes and 2.63 ± 0.80 after 4 h, *P* < 0.05 in all cases vs untreated, n = 8, Figure 7A). Similarly, CX_3_CL1 treatment augmented d-serine level in the medium of mixed glia (1.76 ± 0.19 after 20 minutes, 1.66 ± 0.11 after 2 h and 20 minutes and 1.52 ± 0.24 after 4 h, *P* < 0.05 in all cases vs untreated, n = 8, Figure 7B).

We then tested whether also the A_2A_R agonist VT7 was able to modulate the release of d-serine from glia. For this purpose we stimulated the cells with VT7 (50 nM) in the continuous presence of adenosine deaminase (ADA, 1 U/ml). As for CX_3_CL1, the A_2A_R agonist was able to induce an increment in the basal level of d-serine. Specifically, VT7 treatment produced an increase in d-serine release in both primary purified microglia (up to: 1.28 ± 0.13 after 20 minutes, 1.38 ± 0.10 after 2 h and 20 minutes and 1.60 ± 0.30 after 4 h, *P* < 0.05 in all cases vs untreated, n = 9, Figure 7C) and mixed glia medium (up to: 1.53 ± 0.10 after 20 minutes, 1.37 ± 0.06 after 2 h and 20 minutes and 1.47 ± 0.12 after 4 h, *P* < 0.05 in all cases vs untreated, n = 12, Figure 7D), respectively. Interestingly, the control basal level of d-serine in purified microglia medium was unchanged at different time points, whereas in mixed glia it accumulates upon time, and it had similar values (at 20 minutes, both in M-ACSF or M-ACSF plus ADA, *P* >0.05 for all cases). In addition, the level of basal d-serine released by mixed glia or microglia was in the order of 10 fmol/cell, accordingly to previously reported values [25].

We further tested the ability of VT7 to induce the release of d-serine form neurons in the presence of ADA. During treatment, no variation in the basal level of d-serine was observed, at all time points considered (*P* > 0.05 vs untreated, n = 12). Notably, the basal level of d-serine released by neurons was much lower compared to glia (at 20 minutes: 37.24 ± 7.02 ng/mg of protein, n = 12).

## Discussion

The main finding of the present study is that NMDAR function, in CA1 hippocampal region, is potentiated by CX_3_CL1 application. The activation of the glial A_2A_R and the release of the NMDAR coagonist d-serine are likely major causative factors for the observed NMDA potentiation. Interestingly this effect is postsynaptic in nature, depends on microglia and requires the presence of CX_3_CR1. Specifically, the parameter related to changes in the probability of vesicular release at the synapse, such as PPR, was not affected by CX_3_CL1, indicating that the chemokine did not potentiate NMDA-fEPSPs by altering presynaptic glutamate release (see also [15,44]). In addition, the use of genetically modified mice lacking CX_3_CR1 or inhibiting microglia function by minocycline treatment prevented CX_3_CL1-mediated potentiation of NMDAR function.

### A_2A_R activity is involved in CX_3_CL1 effect

It is established that adenosine acts as neuromodulator of NMDAR function; in particular A_2A_Rs exert a facilitatory role on NMDARs [55,56]. Nevertheless, the consequences of A_2A_R activation on synaptic transmission in physiological conditions of adenosine release remained elusive. Since the ambient level of adenosine is sufficient to activate inhibitory A_1_R but not facilitatory A_2A_R, possibly due to differences in affinity [57], it is believed that specific stimulation such as high-frequency bursts is needed to increase the level of adenosine to values able to activate A_2A_R [58]. Indeed, we have previously demonstrated that stimulation of microglial cells by CX_3_CL1 induces an increase in extracellular level of adenosine [12].

Here, we reported that CX_3_CL1-mediated activation of A_2A_R influences the NMDA-mediated component of excitatory synaptic transmission. In particular, the specific A_2A_R blocker SCH58261, as well as the genetic ablation of A_2A_R, prevented the short-term potentiation of NMDA fEPSPs induced by CX_3_CL1. In addition, the specific A_2A_R agonist VT7 was able to mimic CX_3_CL1 effect, producing a similar potentiation of NMDA-fEPSPs.

### d-serine is essential for CX_3_CL1-mediated potentiation of NMDA-fEPSPs

It is well established that d-serine act as an essential coagonist of NMDAR. The production of d-serine in the mammalian brain results from the activity of SR, an enzyme that converts l-serine into d-serine [59]. Astrocytes synthesize and release d-serine [25], for instance following activation of AMPARs by glutamate released from nerve terminals [24,60]. In contrast, scarce information is available on microglia derived d-serine. It has been reported that microglia express serine racemase [27-29] and release d-serine after activation with amyloid β-peptide [25].

We demonstrated that d-serine is involved in CX_3_CL1-mediated potentiation of NMDA-fEPSPs because its action is prevented by: (i) the selective NMDAR antagonist of the glycine (and d-serine)-binding site DCKA, (ii) the enzymatic degradation of d-serine by DAAO, and (iii) the saturation of the coagonist site by d-serine.

In addition, mass spectrometry analysis revealed that treatment of primary mixed glial cells and purified microglial population with CX_3_CL1 induces a significant increase of d-serine release in the extracellular medium. The glial population used for these studies was derived from cerebral cortices and we are aware that microglial phenotypes differ across brain regions and ages [61,62], so that results from these preparation cannot be directly transferred to hippocampal glial cells; nevertheless these data strongly support a direct role of CX_3_CL1 in controlling d-serine release from glia, that need further investigation, and clearly demonstrate a basal release of d-serine from microglial cells that can be further increased by CX_3_CL1 treatment.

Overall, the evidence suggests that both A_2A_R activity and d-serine release are needed for CX_3_CL1-mediated potentiation of NMDA-fEPSPs. Are these factors interacting in some way? Our results point to a possible crosstalk between A_2A_R activity and d-serine release. First of all, at Schaffer collateral-CA1 synapses the A_2A_R agonist failed to increase NMDA-fEPSPs when the NMDA glycine site was partially blocked by DCKA, indicating an extracellular modulation (via d-serine binding) of NMDAR mediated by the activation of A_2A_R. It is unlikely that the partial block of the NMDAR extracellular site could mask a possible A_2A_R-mediated intracellular modulation of the receptor [63], the latter actually occurring at hippocampal mossy-CA3 synapse via calcium amplification signals [56]. In addition, enzymatic degradation of d-serine by DAAO prevented the A_2A_R agonist mediated potentiation of NMDA-fEPSPs, supporting the d-serine requirement for VT7 effect.

Secondly, by means of mass spectrometry analysis, we have shown that primary glial cells and purified microglia are capable to release d-serine in the medium after stimulation with VT7. These observations suggest a direct role of A_2A_R in controlling the release of d-serine from glia that in turn can modulate NMDAR functions. To the best of our knowledge, this is the first report on adenosine-mediated release of d-serine from glia. A_2A_R are indeed expressed on microglia and astrocytes [49,64,65], usually coupled to G_s_ proteins, enhancing PKA or PKC activity [56,58,66]. Interestingly, PKC activity seems relevant in regulating SR activity and d-serine availability [67-69]. Since it has been reported that neuronal cells can release d-serine, with controversial functions on synaptic NMDAR [70,71], we investigated whether VT7 stimulated d-serine release from neurons. The basal release of d-serine from neurons appeared markedly reduced compared to glia and the activation of neuronal A_2A_R was unable to further increase the extracellular d-serine levels, although we cannot exclude an indirect modulation of neuronal d-serine via other mediators released by CX_3_CL1-activated microglia.

We have previously demonstrated that CX_3_CL1 inhibits AMPA-mediated transmission and LTP expression [15,17] while here we reported that CX_3_CL1 potentiates NMDAR. We speculate that the persistent CX_3_CL1-mediated potentiation of NMDA function and the consequent calcium increase could interfere with the complex mechanisms of LTP induction that depends not only by the precise amplitude but also by the duration of postsynaptic Ca^2+^ increase and by the recent history of synaptic activity. Indeed, it has been demonstrated that prior activation of NMDAR either by synaptic or pharmacological stimulation could effectively inhibit the subsequent LTP induction [72,73].

Since in our experimental setting we recorded synaptic NMDAR blocking AMPAR component, it would be informative to investigate CX_3_CL1-mediated NMDAR modulation in a more physiological condition, where neuronal and glial AMPAR are activable.

Interestingly, the effect of CX_3_CL1 seems to be specific for the synaptic NMDARs that are exclusively gated by the coagonist d-serine [23]. It has been reported that activation of synaptic NMDARs (NR2A-containing NMDARs) preferentially triggers cell survival pathways, whereas stimulation of the whole-cell population of NMDARs and/or selective activation of extrasynaptic NMDARs (NR2B-containing NMDARs) signals toxic stimuli ([74,75], but see also [23]). Therefore, CX_3_CL1-mediated potentiation of synaptic NMDAR function can possibly contribute to the neuroprotective action of CX_3_CL1 described in different *in vitro* and *in vivo* conditions [11,12,14].

**Figure 8:**
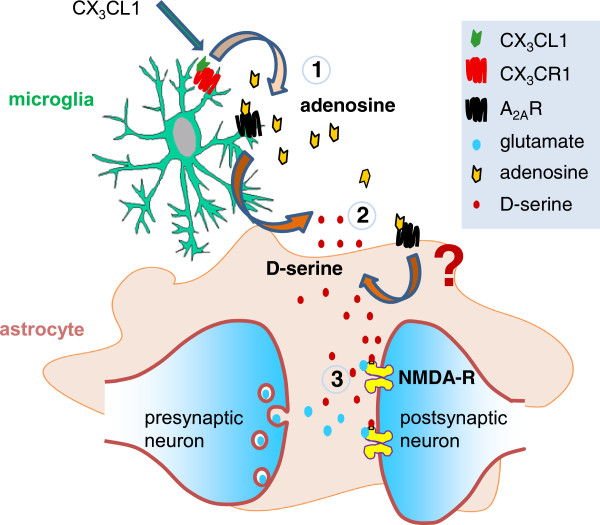
**Suggested mechanism of CX**_**3**_**CL1 action.** Activation of microglial CX_3_CR1 by exogenous application of CX_3_CL1 induces the release of adenosine (1) that in turn activates adenosine receptor type A2 (A_2A_R) located on microglia cells and, possibly, on astrocytes stimulating the release of d-serine (2). The binding of the cofactor d-serine to the synaptically activated *N*-methyl-d-aspartate receptors (NMDARs) (3) potentiates the receptor function. Note that α-amino-3-hydroxy-5-methyl-4-isoxazolepropionic acid receptors (AMPARs) are blocked and, for clarity, only factors demonstrated to play a role in the described mechanism are represented.

Future studies are warranted to discern the role and the precise mechanism of CX_3_CL1-mediated potentiation of NMDA-fEPSPs in both physiological and pathological condition, with particular attention to the sequence of mediators and cell types involved, namely neurons, astrocytes or microglia. Nevertheless, considering overall data, we can speculate that on microglial cells the activation of CX_3_CR1 triggers the release of adenosine that in turn, via A_2A_R activity, increases the release of d-serine from glia leading to a potentiation of NMDA function (Figure 8).

## Conclusions

In summary, we have demonstrated a direct association between the effects of CX_3_CL1 on NMDAR activation and increased levels of d-serine and A_2A_R activity within the CA1 region of the hippocampus. The CX_3_CL1-CX_3_CR1 axis therefore represents a pathway for direct communication between neural cells and microglia.

## Competing interests

The authors declare they have no competing interests.

## Authors’ contributions

LM, MS and CL conceived and designed the experiments; MS and LM performed and analyzed electrophysiological experiments, MADC participated to patch-clamp experiments; MS and GCh performed stimulation experiments with cultures; LA performed mass spectrometry analysis. GCr provided VT7; LM and CL wrote the paper. All authors have read and approved the final version of the manuscript.

## Supplementary Material

Additional file 1: Figure S1 Extracted ion chromatogram detected at m/z 356.1/192.0 (Q1/Q3) which is specific for the Marfey’s derivatives l-Ser (2,4-dinitro-phenyl-5-l-alanine amide- (DNPA-)l-Ser) and d-Ser (DNPA-d-Ser). Note the clear resolution of Marfey’s derivative, DNPA-l-Ser and DNPA-d-Ser. Click here for file

Additional file 2: Figure S2 Extracted ion chromatogram of a blank sample. Note the absence of peaks in modified artificial cerebrospinal fluid (M-ACSF) that interfere with 2,4-dinitro-phenyl-5-l-alanine amide- (DNPA-)l-Ser or DNPA-d-Ser. Click here for file

Additional file 3: Figure S3 CX_3_CL1 does not affect afferent volley and paired pulse facilitation (PPF). **(A)** Representative traces of NMDA-fEPSPs responses in control, after 20 minutes of CX_3_CL1 and after 20 minutes of wash. Note the potentiation of the NMDA-fEPSPs during chemokine treatment without changes in the amplitude of the afferent volley. **(B)** Top: representative traces of *N*-methyl-d-aspartate receptor component of field excitatory postsynaptic potentials (NMDA-fEPSPs) responses evoked by a pair of stimuli (50 ms interval) delivered to the Shaffer collateral in control and after 20 minutes of CX_3_CL1 application, as indicated (vertical scale bar: 0.2 mV, horizontal scale bar: 20 ms). Bottom: histogram of paired-pulse ratio (PPR), expressed as the ratio of the amplitude of the second fEPSP vs the first (n = 6/4). **(C)** Top: representative traces of NMDA currents evoked by a pair of stimuli as in (B); (cells held at 10 mV in standard artificial cerebrospinal fluid (ACSF), vertical scale bar: 20 pA, horizontal scale bar: 20 ms). Bottom: histogram of paired-pulse ratio (PPR) expressed as the ratio of the peak amplitude of the second response vs the first (n = 5/5). Note no difference in PPR before and after CX_3_CL1 application. Bars: mean ± SEM, CX_3_CL1 (5 nM). Click here for file

## References

[B1] Rostène W Kitabgi P Parsadaniantz SM Chemokines: a new class of neuromodulator Nat Rev Neurosci 2007 8 895 903 10.1038/nrn2255 17948033

[B2] Réaux Le Goazigo A Van Steenwinckel J Rostène W Mélik Parsadaniantz S Current status of chemokines in the adult CNS Prog Neurobiol 2013 104 67 92 23454481 10.1016/j.pneurobio.2013.02.001

[B3] Harrison LK Jiang Y Chen S Xia Y Maciejewski D McNamara RK Streit JW Salafranca MN Adhikari S Thompson DA Botti P Bacon KB Feng L Role for neuronally derived fractalkine in mediating interactions between neurons and CX3CR1-expressing microglia Proc Natl Acad Sci USA 1998 95 10896 10901 10.1073/pnas.95.18.10896 9724801 PMC27992

[B4] Hatori K Nagai A Heisel R Ryu JK Kim SU Fractalkine and fractalkine receptors in human neurons and glial cells J Neurosci Res 2002 69 418 426 10.1002/jnr.10304 12125082

[B5] Cardona AE Pioro EP Sasse ME Kostenko V Cardona SM Dijkstra IM Huang D Kidd G Dombrowski S Dutta R Lee JC Cook DN Jung S Lira SA Littman DR Ransohoff RM Control of microglial neurotoxicity by the fractalkine receptor Nat Neurosci 2006 9 917 924 10.1038/nn1715 16732273

[B6] Jung S Aliberti J Graemmel P Sunshine MJ Kreutzberg GW Sher A Littman DR Analysis of fractalkine receptor CX(3)CR1 function by targeted deletion and green fluorescent protein reporter gene insertion Mol Cell Biol 2000 20 4106 4114 10.1128/MCB.20.11.4106-4114.2000 10805752 PMC85780

[B7] Wolf Y Yona S Kim KW Jung S Microglia, seen from the CX3CR1 angle Front Cell Neurosci 2013 7 26 23507975 10.3389/fncel.2013.00026 PMC3600435

[B8] Hughes PM Botham MS Frentzel S Mir A Perry VH Expression of fractalkine (CX3CL1) and its receptor, CX3CR1, during acute and chronic inflammation in the rodent CNS Glia 2002 37 314 327 10.1002/glia.10037 11870871

[B9] Sunnemark D Eltayeb S Nilsson M Wallström E Lassmann H Olsson T CX3CL1 (fractalkine) and CX3CR1 expression in myelin oligodendrocyte glycoprotein-induced experimental autoimmune encephalomyelitis: kinetics and cellular origin J Neuroinflammation 2005 2 17 31 10.1186/1742-2094-2-17 16053521 PMC1188067

[B10] D'Haese JG Friess H Ceyhan GO Therapeutic potential of the chemokine-receptor duo fractalkine/CX3CR1: an update Expert Opin Ther Targets 2012 16 613 618 10.1517/14728222.2012.682574 22530606

[B11] Limatola C Lauro C Catalano M Ciotti MT Bertollini C Di Angelantonio S Ragozzino D Eusebi F Chemokine CX3CL1 protects rat hippocampal neurons against glutamate-mediated excitotoxicity J Neuroimmunol 2005 166 19 28 10.1016/j.jneuroim.2005.03.023 16019082

[B12] Lauro C Di Angelantonio S Cipriani R Sobrero F Antonilli L Brusadin V Ragozzino D Limatola C Activity of adenosine receptors type 1 is required for CX3CL1-mediated neuroprotection and neuromodulation in hippocampal neurons J Immunol 2008 180 7590 7596 18490761 10.4049/jimmunol.180.11.7590

[B13] Lauro C Cipriani R Catalano M Trettel F Chece G Brusadin V Antonilli L van Rooijen N Eusebi F Fredholm BB Limatola C Adenosine A1 receptors and microglial cells mediate CX3CL1-induced protection of hippocampal neurons against Glu-induced death Neuropsychopharmacology 2010 35 1550 1559 10.1038/npp.2010.26 20200508 PMC3055460

[B14] Cipriani R Villa P Chece G Lauro C Paladini A Micotti E Perego C De Simoni MG Fredholm BB Eusebi F Limatola C CX3CL1 is neuroprotective in permanent focal cerebral ischemia in rodents J Neurosci 2011 31 16327 16335 10.1523/JNEUROSCI.3611-11.2011 22072684 PMC6633249

[B15] Ragozzino D Di Angelantonio S Trettel F Bertollini C Maggi L Gross C Charo IF Limatola C Eusebi F Chemokine fractalkine/CX_3_CL1 negatively modulates active glutamatergic synapses in rat hippocampal neurons J Neurosci 2006 26 10488 10498 10.1523/JNEUROSCI.3192-06.2006 17035533 PMC6674698

[B16] Bertollini C Ragozzino D Gross C Limatola C Eusebi F Fractalkine/CX3CL1 depresses central synaptic transmission in mouse hippocampal slices Neuropharmacology 2006 51 816 821 10.1016/j.neuropharm.2006.05.027 16815480

[B17] Maggi L Trettel F Scianni M Bertollini C Eusebi F Fredholm BB Limatola C LTP impairment by fractalkine/CX(3)CL1 in mouse hippocampus is mediated through the activity of adenosine receptor type 3 (A(3)R) J Neuroimmunol 2009 215 36 42 10.1016/j.jneuroim.2009.07.016 19709758

[B18] MacDermott AB Mayer ML Westbrook GL Smith SJ Barker JL NMDA-receptor activation increases cytoplasmic calcium concentration in cultured spinal cord neurones Nature 1986 321 519 522 10.1038/321519a0 3012362

[B19] Cull-Candy S Brickley S Farrant M NMDA receptor subunits: diversity, development and disease Curr Opin Neurobiol 2001 11 327 335 10.1016/S0959-4388(00)00215-4 11399431

[B20] Hollmann M Heinemann S Cloned glutamate receptors Annu Rev Neurosci 1994 17 31 108 10.1146/annurev.ne.17.030194.000335 8210177

[B21] Johnson JW Ascher P Glycine potentiates the NMDA response in cultured mouse brain neurons Nature 1987 325 529 531 10.1038/325529a0 2433595

[B22] Matsui T Sekiguchi M Hashimoto A Tomita U Nishikawa T Wada K Functional comparison of d-serine and glycine in rodents: the effect on cloned NMDA receptors and the extracellular concentration J Neurochem 1995 65 454 458 7790891 10.1046/j.1471-4159.1995.65010454.x

[B23] Papouin T Ladépêche L Ruel J Sacchi S Labasque M Hanini M Groc L Pollegioni L Mothet JP Oliet SH Synaptic and extrasynaptic NMDA receptors are gated by different endogenous coagonists Cell 2012 150 633 646 10.1016/j.cell.2012.06.029 22863013

[B24] Schell MJ Brady RO Jr Molliver ME Snyder SH d-serine as a neuromodulator: regional and developmental localization in rat brain glia resemble NMDA receptors J Neurosci 1997 17 1604 1615 9030620 10.1523/JNEUROSCI.17-05-01604.1997 PMC6573391

[B25] Wu SZ Bodles AM Porter MM Griffin WS Basile AS Barger SW Induction of serine racemase expression and d-serine release from microglia by amyloid beta-peptide J Neuroinflammation 2004 1 2 10.1186/1742-2094-1-2 15285800 PMC483052

[B26] Williams SM Diaz CM Macnab LT Sullivan RK Pow DV Immunocytochemical analysis of d-serine distribution in the mammalian brain reveals novel anatomical compartmentalizations in glia and neurons Glia 2006 53 401 411 10.1002/glia.20300 16342169

[B27] Sasabe J Chiba T Yamada M Okamoto K Nishimoto I Matsuoka M Aiso S d-serine is a key determinant of glutamate toxicity in amyotrophic lateral sclerosis EMBO J 2007 26 4149 5159 10.1038/sj.emboj.7601840 17762863 PMC2230675

[B28] Wang W Barger SW Cross-linking of serine racemase dimer by reactive oxygen species and reactive nitrogen species J Neurosci Res 2012 90 1218 1229 10.1002/jnr.22832 22354542 PMC3323679

[B29] Hayashi Y Ishibashi H Hashimoto K Nakanishi H Potentiation of the NMDA receptor-mediated responses through the activation of the glycine site by microglia secreting soluble factors Glia 2006 53 660 668 10.1002/glia.20322 16498631

[B30] Block ML Zecca L Hong JS Microglia-mediated neurotoxicity: uncovering the molecular mechanisms Nat Rev Neurosci 2007 8 57 69 10.1038/nrn2038 17180163

[B31] Flavin MP Zhao G Ho LT Microglial tissue plasminogen activator (tPA) triggers neuronal apoptosis *in vitro* Glia 2000 29 347 354 10.1002/(SICI)1098-1136(20000215)29:4<347::AID-GLIA5>3.0.CO;2-8 10652444

[B32] Jarvis CR Xiong ZG Plant JR Churchill D Lu WY MacVicar BA MacDonald JF Neurotrophin modilation of NMDA receptors in cultures murine and isolated rat neurons J Neurophysiol 1997 78 2363 2371 9356388 10.1152/jn.1997.78.5.2363

[B33] Nakanishi H Microglial functions and proteases Mol Neurobiol 2003 27 163 176 10.1385/MN:27:2:163 12777686

[B34] Yang S Liu ZW Qiao HF Zhou WX Zhang YX Interleukin-1 b enhances NMDA receptor-mediated current but inhibits excitatory transmission Brain Res 2005 1034 172 179 10.1016/j.brainres.2004.11.018 15713269

[B35] Deiva K Geeraerts T Salim H Leclerc P Héry C Hugel B Freyssinet JM Tardieu M Fractalkine reduces *N*-methyl-d-aspartate-induced calcium flux and apoptosis in human neurons through extracellular signal-regulated kinase activation Eur J Neurosci 2004 20 3222 3232 10.1111/j.1460-9568.2004.03800.x 15610155

[B36] Cook DN Cook DN Chen SC Sullivan LM Manfra DJ Wiekowski MT Prosser DM Vassileva G Lira SA Generation and analysis of mice lacking the chemokine fractalkine Mol Cell Biol 2001 21 3159 3165 10.1128/MCB.21.9.3159-3165.2001 11287620 PMC86945

[B37] Johansson B Halldner L Dunwiddie TV Masino SA Poelchen W Gimenez-Llort L Escorihuela RM Fernandez-Teruel A Wiesenfeld-Hallin Z Xu XJ Hardemark A Betsholtz C Herlenius E Fredholm BB Hyperalgesia, anxiety, and decreased hypoxic neuroprotection in mice lacking the adenosine A1 receptor Proc Natl Acad Sci USA 2001 98 9407 9412 10.1073/pnas.161292398 11470917 PMC55434

[B38] Salvatore CA Tilley SL Latour AM Fletcher DS Koller BH Jacobson MA Disruption of the A(3) adenosine receptor gene in mice and its effect on stimulated inflammatory cells J Biol Chem 2000 275 4429 4434 10.1074/jbc.275.6.4429 10660615

[B39] Chen JF Huang Z Ma J Zhu J Moratalla R Standaert D Moskowitz MA Fink JS Schwarzschild MA A(2A) adenosine receptor deficiency attenuates brain injury induced by transient focal ischemia in mice J Neurosci 1999 19 9192 9200 10531422 10.1523/JNEUROSCI.19-21-09192.1999 PMC6782932

[B40] Berna MJ Ackermann BL Quantification of serine enantiomers in rat brain microdialysate using Marfey’s reagent and LC/MS/MS J Chromatogr 2007 846 359 363 10.1016/j.jchromb.2006.08.029 16962391

[B41] Neagu B Strominger NL Carpenter DO Contribution of NMDA receptor-mediated component to the EPSP in mouse Schaffer collateral synapses under single pulse stimulation protocol Brain Res 2008 1240 54 61 18817765 10.1016/j.brainres.2008.09.007

[B42] Zucker RS Short-term synaptic plasticity Ann Rev Neurosci 1989 12 13 31 10.1146/annurev.ne.12.030189.000305 2648947

[B43] Maggi L Sola E Minneci F Le Magueresse C Changeux JP Cherubini E Persistent decrease in synaptic efficacy induced by nicotine at Schaffer collateral-CA1 synapses in the immature rat hippocampus J Physiol 2004 559 863 874 10.1113/jphysiol.2004.067041 15272042 PMC1665176

[B44] Maggi L Scianni M Branchi I D’Andrea I Lauro C Limatola C CX(3)CR1 deficiency alters hippocampal-dependent plasticity phenomena blunting the effects of enriched environment Front Cell Neurosci 2011 5 22 22025910 10.3389/fncel.2011.00022 PMC3198035

[B45] Suzuki H Sugimura Y Iwama S Suzuki H Nobuaki O Nagasaki H Minocycline prevents osmotic demyelination syndrome by inhibiting the activation of microglia J Am Soc Nephrol 2010 21 2090 2098 10.1681/ASN.2010040438 21030598 PMC3014022

[B46] Yrjanheikki J Keinanen R Pellikka M Hokfelt T Koistinaho J Tetracyclines inhibit microglial activation and are neuroprotective in global brain ischemia Proc Natl Acad Sci USA 1998 95 15769 10.1073/pnas.95.26.15769 9861045 PMC28119

[B47] Tikka TM Koistinaho JE Minocycline provides neuroprotection against *N*-methyl-d-aspartate neurotoxicity by inhibiting microglia J Immunol 2001 166 7527 7533 11390507 10.4049/jimmunol.166.12.7527

[B48] Piccinin S Di Angelantonio S Piccioni A Volpini R Cristalli G Fredholm BB Limatola C Eusebi F Ragozzino D CX3CL1-induced modulation at CA1 synapses reveals multiple mechanisms of EPSC modulation involving adenosine receptor subtypes J Neuroimmunol 2010 224 85 92 10.1016/j.jneuroim.2010.05.012 20570369

[B49] Sebastião AM Ribeiro JA Tuning and fine-tuning of synapses with adenosine Curr Neuropharmacol 2009 7 180 194 10.2174/157015909789152128 20190960 PMC2769002

[B50] Lopes LV Cunha RA Kull B Fredholm BB Ribeiro JA Adenosine A_2A_ receptor facilitation of hippocampal synaptic transmission is dependent on tonic A_1_ receptor inhibition Neuroscience 2002 112 319 329 10.1016/S0306-4522(02)00080-5 12044450

[B51] Miller RF d-serine as a glial modulator of nerve cells Glia 2004 47 275 283 10.1002/glia.20073 15252817

[B52] Wolosker H Blackshaw S Snyder SH Serine racemase: a glial enzyme synthesizing d-serine to regulate glutamate-*N*-methyl-d-aspartate neurotransmission Proc Natl Acad Sci USA 1999 96 13409 13414 10.1073/pnas.96.23.13409 10557334 PMC23961

[B53] Molla G Sacchi S Bernasconi M Pilone MS Fukui K Polegioni L Characterization of human d-amino acid oxidase FEBS Lett 2006 580 2358 2364 10.1016/j.febslet.2006.03.045 16616139

[B54] Henneberger C Papouin T Oliet SH Rusakov DA Long-term potentiation depends on release of d-serine from astrocytes Nature 2010 463 232 236 10.1038/nature08673 20075918 PMC2807667

[B55] Tebano MT Martire M Rebola N Pepponi R Domenici MR Gro MC Schwarzschild MA Chen JF Cunha RA Popoli P Adenosine A2A receptors and mGluR5 are co-localized and functionally interact in the hippocampus: a possible key mechanism in the modulation of *N*-methyl-d-aspartate effects J Neurochem 2005 95 1188 1200 10.1111/j.1471-4159.2005.03455.x 16271052

[B56] Rebola N Lujan R Cunha RA Mulle C Long-term potentiation of NMDA-EPSCs at hippocampal mossy fiber synapses: an essential role for adenosine A2A receptors Neuron 2008 57 121 134 10.1016/j.neuron.2007.11.023 18184569

[B57] Ciruela F Casado V Rodrigues RJ Lujan R Burgueno J Canals M Borycz J Rebola N Goldberg SR Mallol J Presynaptic control of striatal glutamatergic neurotransmission by adenosine A1-A2A receptor heteromers J Neurosci 2006 26 2080 2087 10.1523/JNEUROSCI.3574-05.2006 16481441 PMC6674939

[B58] Cunha RA Adenosine as a neuromodulator and as a homeostatic regulator in the nervous system: different roles, different sources and different receptors Neurochem Int 2001 38 107 125 10.1016/S0197-0186(00)00034-6 11137880

[B59] Mustafa AK Kim PM Snyder SH d-serine as a putative glial neurotransmitter Neuron Glia Biol 2004 1 275 281 16543946 10.1017/S1740925X05000141 PMC1403160

[B60] Mothet JP Pollegioni L Ouanounou G Martineau M Fossier P Baux G Glutamate receptor activation triggers a calcium-dependent and SNARE protein-dependent release of the gliotransmitter d-serine Proc Natl Acad Sci USA 2005 102 5606 5611 10.1073/pnas.0408483102 15800046 PMC556243

[B61] Lawson LJ Perry VH Dri P Gordon S Heterogeneity in the distribution and morphology of microglia in the normal adult mouse brain Neuroscience 1990 39 151 170 10.1016/0306-4522(90)90229-W 2089275

[B62] Njie EG Boelen E Stassen FR Steinbusch HW Borchelt DR Streit WJ *Ex vivo* cultures of microglia from young and aged rodent brain reveal age-related changes in microglial function Neurobiol Aging 2012 33 195.e1 12 10.1016/j.neurobiolaging.2010.05.008 20580465 PMC4162517

[B63] Lu WY Jackson MF Bai D Orser BA MacDonald JF In CA1 pyramidal neurons of the hippocampus protein kinase C regulates calcium-dependent inactivation of NMDA receptors J Neurosci 2000 20 4452 4461 10844014 10.1523/JNEUROSCI.20-12-04452.2000 PMC6772451

[B64] Rebola N Simões AP Canas PM Tomé AR Andrade GM Barry CE Agostinho PM Lynch MA Cunha RA Adenosine A2A receptors control neuroinflammation and consequent hippocampal neuronal dysfunction J Neurochem 2011 117 100 111 10.1111/j.1471-4159.2011.07178.x 21235574

[B65] Cristóvão-Ferreira S Navarro G Brugarolas M Pérez-Capote K Vaz SH Fattorini G Conti F Lluis C Ribeiro JA McCormick PJ Casadó V Franco R Sebastião AM Modulation of GABA transport by adenosine A1R-A2AR heteromers, which are coupled to both Gs- and G(i/o)-proteins J Neurosci 2011 31 15629 15639 10.1523/JNEUROSCI.2526-11.2011 22049406 PMC6623011

[B66] Fredholm BB IJzerman AP Jacobson KA Klotz KN Linden J International Union of Pharmacology. XXV. Nomenclature and classification of adenosine receptors Pharmacol Rev 2001 53 527 552 11734617 PMC9389454

[B67] Martineau M Baux G Mothet JP d-serine signalling in the brain: friend and foe Trends Neurosci 2006 29 481 491 10.1016/j.tins.2006.06.008 16806506

[B68] Vargas Lopes C Madeira C Kahn SA Albino Do Couto I Bado P Houzel JC De Miranda J De Freitas MS Ferreira ST Panizzutti R Protein kinase C activity regulates d-serine availability in the brain J Neurochem 2011 116 281 290 10.1111/j.1471-4159.2010.07102.x 21070240

[B69] Fuchs SA Berger R de Koning TJ d-serine: the right or wrong isoform? Brain Res 2011 1401 104 117 21676380 10.1016/j.brainres.2011.05.039

[B70] Rosenberg D Artoul S Segal AC Kolodney G Radzishevsky I Dikopoltsev E Foltyn VN Inoue R Mori H Billard JM Wolosker H Neuronal d-serine and glycine release via the Asc-1 transporter regulates NMDA receptor-dependent synaptic activity J Neurosci 2013 In press 10.1523/JNEUROSCI.3836-12.2013 PMC6619521 23426681

[B71] Wolosker H NMDA receptor regulation by d-serine: new findings and perspectives Mol Neurobiol 2007 36 152 164 10.1007/s12035-007-0038-6 17952659

[B72] Huang YY Colino A Selig DK Malenka RC The influence of prior synaptic activity on the induction of long-term potentiation Science 1992 255 730 733 10.1126/science.1346729 1346729

[B73] Izumi Y Clifford DB Zorumski CF Low concentrations of *N*-methyl-d-aspartate inhibit the induction of long-term potentiation in rat hippocampal slices Neurosci Lett 1992 137 245 248 10.1016/0304-3940(92)90414-3 1350078

[B74] Soriano FX Hardingham GE Compartmentalized NMDA receptor signalling to survival and death J Physiol 2007 584 381 387 10.1113/jphysiol.2007.138875 17690142 PMC2277150

[B75] Leveille F El Gaamouch F Gouix E Lecocq M Lobner D Nicole O Buisson A Neuronal viability is controlled by a functional relation between synaptic and extrasynaptic NMDA receptors FASEB J 2008 22 4258 4271 10.1096/fj.08-107268 18711223

