# Supplementary figures and images for: Fractalkine (CX3CL1) enhances hippocampal N-methyl-d-aspartate receptor (NMDAR) function via d-serine and adenosine receptor type A2 (A2AR) activity
Source: J Neuroinflammation. 2013 Aug 27;10:108. doi: 10.1186/1742-2094-10-108 (PMC3765929; doi:10.1186/1742-2094-10-108)

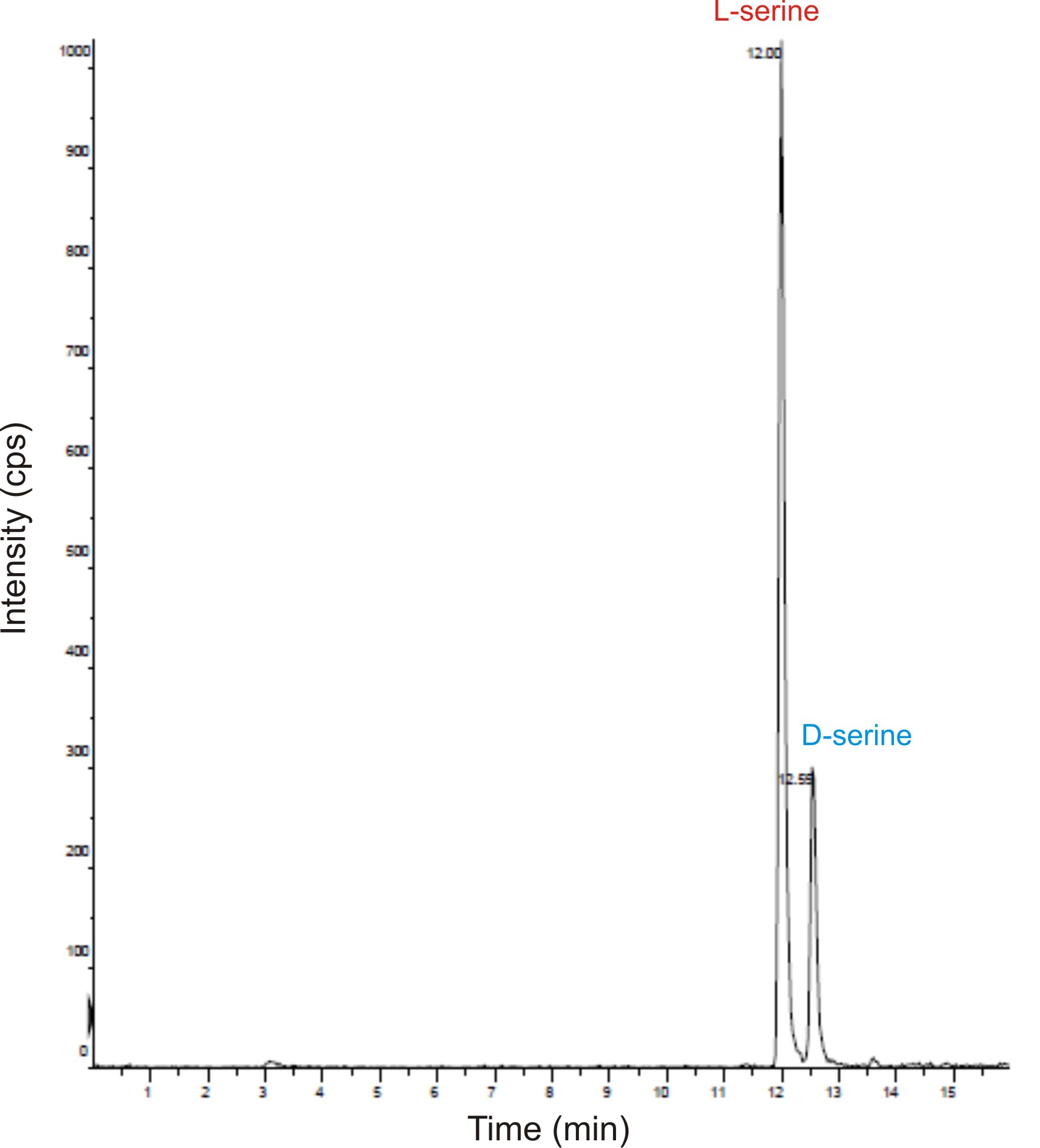

Supplement: Additional file 1: Figure S1 — Extracted ion chromatogram detected at m/z 356.1/192.0 (Q1/Q3) which is specific for the Marfey’s derivatives l-Ser (2,4-dinitro-phenyl-5-l-alanine amide- (DNPA-)l-Ser) and d-Ser (DNPA-d-Ser). Note the clear resolution of Marfey’s derivative, DNPA-l-Ser and DNPA-d-Ser. [file 1742-2094-10-108-S1.jpeg]

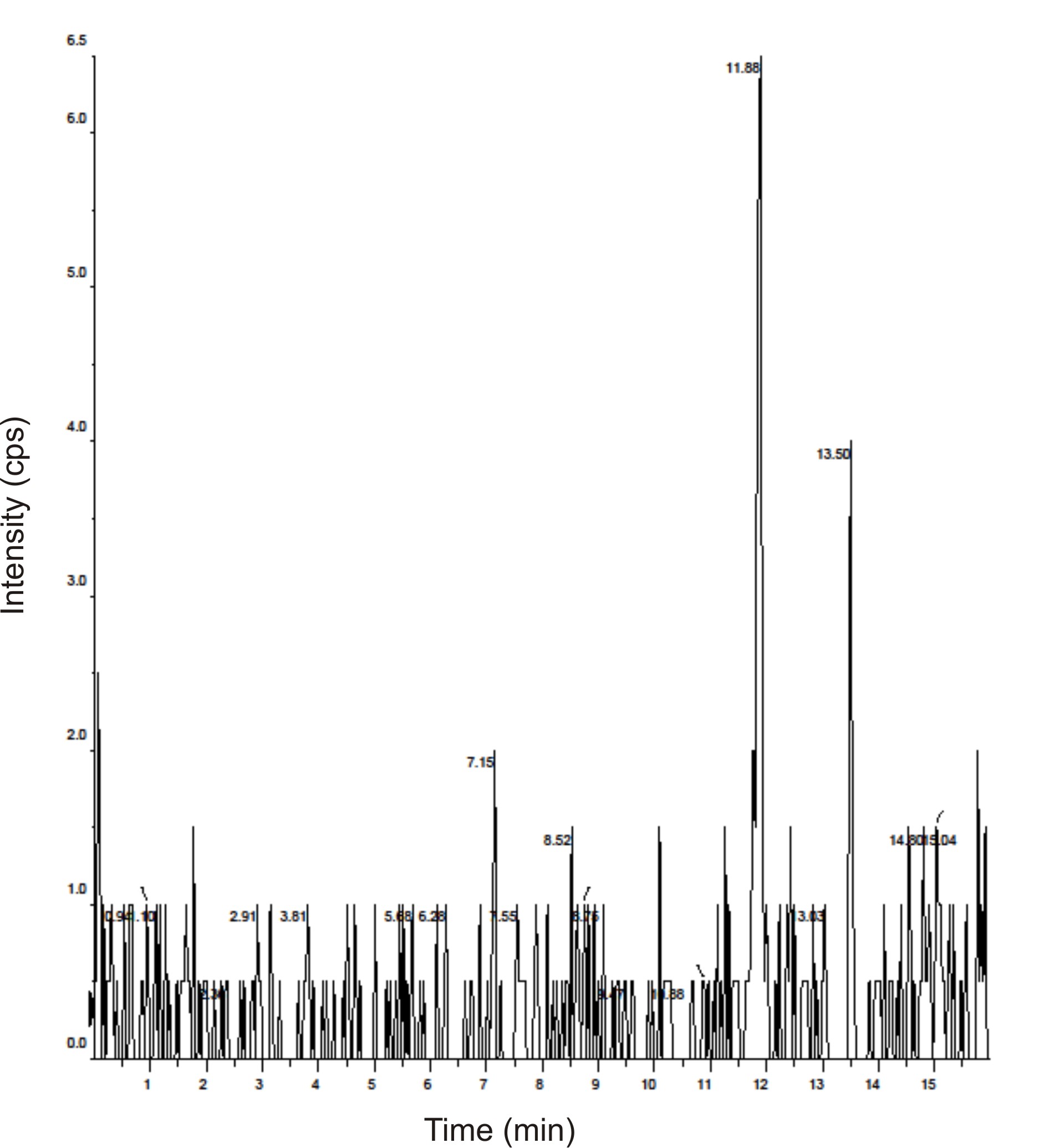

Supplement: Additional file 2: Figure S2 — Extracted ion chromatogram of a blank sample. Note the absence of peaks in modified artificial cerebrospinal fluid (M-ACSF) that interfere with 2,4-dinitro-phenyl-5-l-alanine amide- (DNPA-)l-Ser or DNPA-d-Ser. [file 1742-2094-10-108-S2.jpeg]

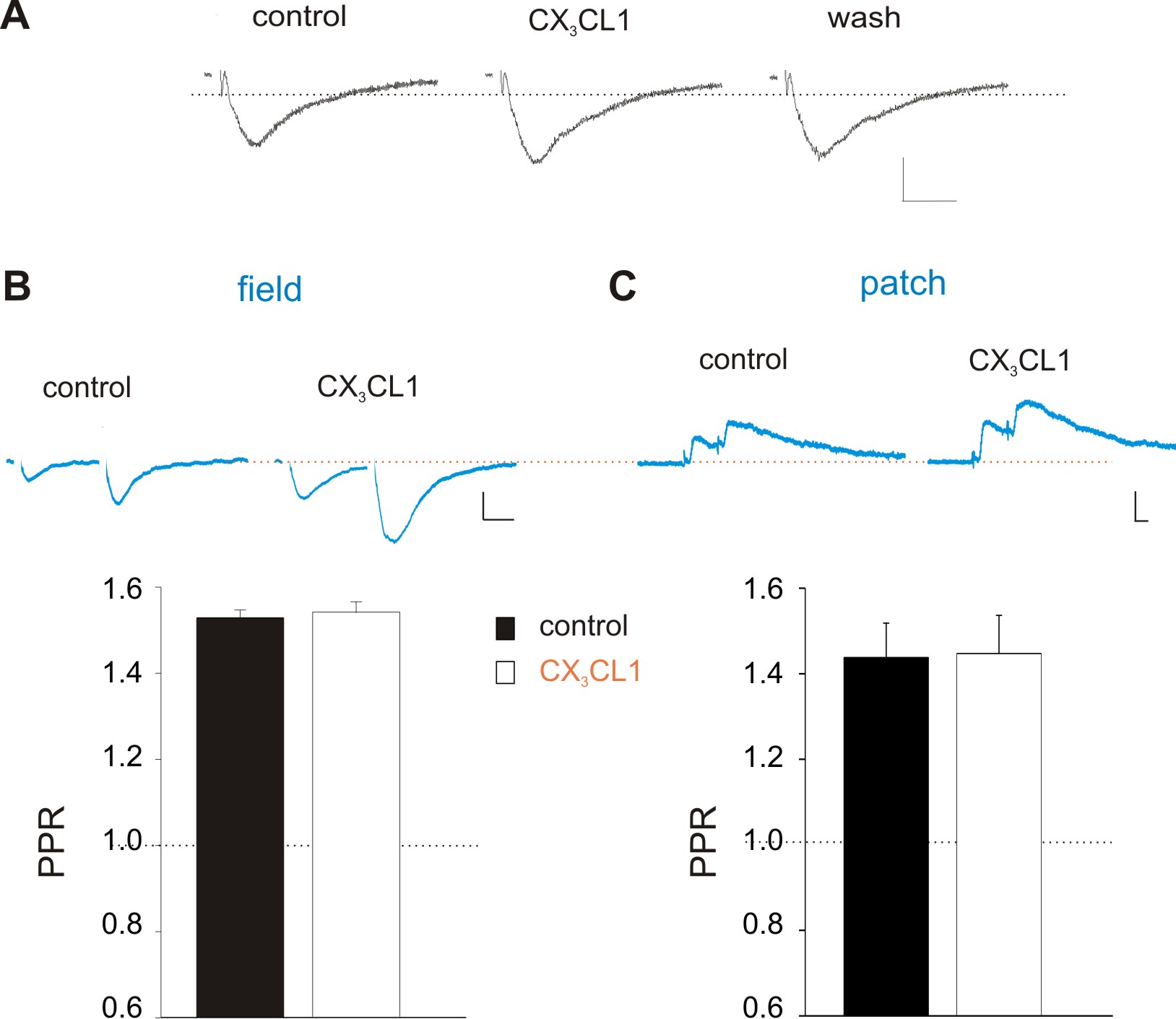

Supplement: Additional file 3: Figure S3 — CX3CL1 does not affect afferent volley and paired pulse facilitation (PPF). (A) Representative traces of NMDA-fEPSPs responses in control, after 20 minutes of CX3CL1 and after 20 minutes of wash. Note the potentiation of the NMDA-fEPSPs during chemokine treatment without changes in the amplitude of the afferent volley. (B) Top: representative traces of N-methyl-d-aspartate receptor component of field excitatory postsynaptic potentials (NMDA-fEPSPs) responses evoked by a pair of stimuli (50 ms interval) delivered to the Shaffer collateral in control and after 20 minutes of CX3CL1 application, as indicated (vertical scale bar: 0.2 mV, horizontal scale bar: 20 ms). Bottom: histogram of paired-pulse ratio (PPR), expressed as the ratio of the amplitude of the second fEPSP vs the first (n = 6/4). (C) Top: representative traces of NMDA currents evoked by a pair of stimuli as in (B); (cells held at 10 mV in standard artificial cerebrospinal fluid (ACSF), vertical scale bar: 20 pA, horizontal scale bar: 20 ms). Bottom: histogram of paired-pulse ratio (PPR) expressed as the ratio of the peak amplitude of the second response vs the first (n = 5/5). Note no difference in PPR before and after CX3CL1 application. Bars: mean ± SEM, CX3CL1 (5 nM). [file 1742-2094-10-108-S3.jpeg]
